# A systematic review of peer support interventions for student mental health and well-being in higher education

**DOI:** 10.1192/bjo.2023.603

**Published:** 2023-12-15

**Authors:** Julia Pointon-Haas, Luqmaan Waqar, Rebecca Upsher, Juliet Foster, Nicola Byrom, Jennifer Oates

**Affiliations:** Institute of Psychiatry, Psychology and Neuroscience, Department of Psychology, King's College London, UK; School of Health Sciences, Faculty of Health and Medical Sciences, University of Surrey, UK

**Keywords:** Peer support, well-being, mental health, university, higher education

## Abstract

**Background:**

Higher education institutions (HEIs) are seeking effective ways to address the rising demand for student mental health services. Peer support is widely considered a viable option to increase service capacity; however, there are no agreed definitions of peer support, making it difficult to establish its impact on student mental health and well-being.

**Aims:**

This systematic review aims to better understand and evaluate peer support in HEIs.

**Method:**

Five databases, OpenGrey and Grey Matters were searched in May 2021. Included studies were quantitative, longitudinal (with and without a control) or cross-sectional with a control. The vote-counting method was used for synthesis. The risk of bias was assessed with the National Institutes of Health Quality Assessment Tool.

**Results:**

Three types of peer support were represented in 28 papers: peer-led support groups, peer mentoring and peer learning. Peer learning and peer mentoring had more positive, significant results reported for the outcomes of anxiety and stress. Peer-led support groups were the only type targeting students with mental health difficulties.

**Conclusions:**

The heterogeneity of measures and outcomes prevents firm conclusions on the effectiveness of peer support for mental health and well-being. Most studies were rated ‘poor’ or ‘fair’ in their risk of bias. There is not a solid evidence base for the effectiveness of peer support. Nonetheless, HEIs can use the terminology developed in this review for shared discussions that guide more robust research and evaluation of peer support as an intervention.

There are growing concerns for students’ mental health in higher education,^1^ with significant numbers of students reporting distress.^2^ Higher education institutions (HEIs) refer to any tertiary education leading to an academic degree award.^3^ In the World Health Organization's international college student survey, a third of first-year students screened positive for at least one common anxiety, mood or substance use disorder as defined by the DSM-IV.^4^ Correspondingly, British HEIs reported a 94% increase in demand for counselling services from 2012 to 2017.^5^ Despite service demand rising, only 4.9% of students disclosed a mental health condition to their HEI as a disability in the 2019–2020 enrolment,^6^ indicating that barriers to student help-seeking still exist. HEIs are seeking effective ways to support students, considering the increased demand and low disclosure rates. Globally, a settings-based, whole-systems approach to improving health has been widely advocated for.^7–10^ In UK HEIs, this has gained momentum with the ‘University Mental Health Charter’, which outlines how institutions can take a ‘whole-university’ approach to mental health and encourages peer support to be represented in their strategies.^11^

Peer support is ‘support provided by and for people with similar conditions, problems or experiences’.^12^ It can be delivered in various ways, including one-to-one mentoring and self-help groups.^13^ Convening people with similar experiences creates a supportive space underpinned by respect, collective responsibility and an agreement on what is helpful.^14^ Two approaches exist: informal and formal. Informal peer support happens naturally within communities when people help others in similar circumstances based on their lived experience.^12^ Without structure, this form of peer support is challenging to evaluate. In contrast, formal peer support brings people with similar experiences together intentionally to share knowledge for mutual benefit, building social connection and reducing loneliness.^13,15^ Formal peer support will be the focus of this review, with the term generally describing higher education students helping each other based on their common lived experience of being a student.

Students find peer support easy to use, and recent research suggests it can increase support service accessibility.^16^ Students disclose more to peers than to their HEIs: 75% of students who experienced mental health difficulties reported telling a peer.^17^ Since students prefer seeking help from friends more than professional services,^18,19^ HEIs want to harness this natural preference through peer support, as recommended in the University Mental Health Charter.^11^ A quantitative meta-analysis of 23 peer-run programmes for depression in community health settings found that the interventions produced significant reductions in depressive symptoms, performing as well as professional-led interventions and significantly better than no treatment.^20^ Although peer support is used by many and seems promising, its effectiveness in higher education settings is unknown.^21^

There is currently no comprehensive quantitative review of the published and grey literature on peer support interventions evaluated in higher education settings. Peer support in clinical settings is well defined, with competency standards and fidelity assessments providing an emerging standard of practice.^22^ In contrast, different forms of peer support exist in HEIs, and guidance is still needed to delineate between models.^23^ Limited search terms in a previous systematic review,^21^ which included only three studies, missed relevant research on other forms of peer support. Although studies outline individual benefits for specific types of peer support in higher education settings,^24–27^ no current reviews collate all forms of peer support in HEIs that target mental health and well-being in the literature.

Defining a ‘peer’ is also critical to understanding how the kinds of peer support in higher education differ. In broader contexts, definitions of a peer most commonly refer to those who have lived experience with mental health difficulties or have used mental health services in clinical settings.^28^ In HEIs, however, other identities, such as ethnicity, sexual orientation or course of study, may provide an additional point of connection. For example, research recommends creating more peer support spaces for Black students.^29,30^ A synthesis of the definitions of peer support and what it means to be a peer are needed to inform and evaluate current practice, direct future research and clarify the role of peer support in a whole-university approach to student mental health and well-being.

The aim of this review was to screen relevant literature on peer support interventions evaluated in higher education settings worldwide, to identify current practice and assess its effect on measures of student mental health and well-being, by undertaking the following objectives: (a) to synthesise and categorise types of peer support and define peers according to study characteristics; and (b) to evaluate the effectiveness of peer support in higher education for improving student mental health and well-being according to the developed intervention categories.

For the purpose of this review, mental health and well-being are defined according to the University Mental Health Charter. Mental health refers to ‘a full spectrum of experiences ranging from good mental health to mental illness’ and well-being encompasses ‘a wider framework, of which mental health is an integral part, but which also includes physical and social wellbeing’.^11^

## Method

The systematic review protocol was registered with the International Prospective Register of Systematic Reviews (PROSPERO; identifier: CRD42021256552). No amendments were made. The review followed the Preferred Reporting Items for Systematic Reviews and Meta-Analyses (PRISMA)^31,32^ and Synthesis Without Meta-Analysis (SWiM) guidance.^33^

### Eligibility criteria

Studies with a quantitative longitudinal design were included, with and without a control, or comparator. Cross-sectional studies with a control condition were included. Cross-sectional studies lacking a control were excluded. Qualitative-only studies were excluded. Any students (aged ≥18 years) in HEIs were included. Interventions delivering peer support in higher education were included. Interventions that provided a one-off psychoeducation initiative were excluded.

Studies with and without a comparator, or control, were included. Comparator conditions included those not participating in peer support, a waitlist, informal groups, website access only, year group or faculty mentoring. Where a study used a comparator, the population had to be from a similar higher education setting as the primary intervention.

The outcome of this review was a change in the quantitative measure of well-being or mental health for HEI students, such as stress, anxiety, depression, well-being, loneliness and belonging. Studies were excluded if no quantitative measures were reported. Outcomes for anyone other than students receiving the peer support intervention were excluded.

### Information sources

In May 2021, a worldwide systematic search of studies written in English was conducted in the databases: Ovid (PsycINFO, Medline, EMBASE), Web of Science (Core Collection) and the Education Resources Information Center (ERIC). The search was limited to the past 30 years in alignment with a previous review that included a study from 1991.^20^ Grey literature was searched for through OpenGrey^34^ and Grey Matters.^35^

### Search strategy

Search terms were developed in PsycINFO and adapted for other databases. Key words included population terms (e.g. ‘university’ or ‘student’), intervention terms (e.g. ‘peer support’, ‘peer mentoring’ or ‘peer-assisted learning’ or ‘peer to peer’ or ‘peer tutoring’ or ‘peer health education’) and outcome terms (e.g. ‘mental health’ or ‘well-being’). A complete search strategy (see Supplementary Table 1 available at https://doi.org/10.1192/bjo.2023.603) was developed with existing systematic reviews with similar keywords, to identify relevant MeSH and free-text terms.^21,36,37^ Free-text terms identified in relevant studies from a scoping review were also included (e.g.^23–26^). Grey literature was identified through OpenGrey^34^ and Grey Matters,^35^ a scoping review and backward citation tracking of included full-text studies. Authors were contacted during the search process via email for clarification or full-text articles.

### Selection process

In stage 1, titles and abstracts of papers identified by electronic searches were exported to the Windows desktop version of Clarivate EndNote 20 (London, UK; see https://endnote.com/downloads) from all databases, to remove duplicates.^38^ The citations were then exported using a Windows browser with the web-based software as a service application, ‘Rayyan-intelligent systematic review’ (Qatar Computing Reseach Institute, Boston, USA; see www.rayyan.ai), where independent screening by two researchers was conducted.^39^ The lead reviewer (J.P.-H.) screened all titles and abstracts, and the second researcher (L.W.) screened 50%. If there was any uncertainty at this stage, papers were included for full-text review. In stage 2, full texts of all papers included in stage 1 were independently screened for inclusion by both researchers (J.P.-H. and L.W.). Any discrepancies were resolved by a third researcher (J.F.).

### Data collection process

Data extraction was managed in Windows Microsoft Excel (version 2309) with tables (e.g. study characteristics) and figures (e.g. risk-of-bias data) created. The team developed and approved a data extraction form before being piloted on five studies independently by two researchers (J.P.-H. and L.W.). Data extraction for these studies was compared and refined before applying it to all included studies.

### Data items

The following data items were extracted upon availability and reported:

- Publication characteristics: year of publication, country and HEI of recruitment;
- Methodology and study design: longitudinal or cross-sectional with a control;
- Population characteristics: sample size, attrition, the mental health status of the population, level of study, students’ year of study, gender, mean age and ethnicity;
- Intervention characteristics: type and objective of peer support, number of peer support sessions, duration of intervention, format of delivery and who the peer support is for;
- Outcome characteristics/measures: quantitative measures of well-being and/or mental health at pre- and post-intervention for longitudinal studies (with or without a control) or at a particular time point with a control for cross-sectional studies;
- Results: mean and standard deviation at baseline and follow-up, *P*-value and confidence intervals from the intervention group and comparator (where applicable).

Missing data was denoted as ‘not reported’ to indicate its absence for the risk-of-bias assessment.

### Study risk-of-bias assessment

The methodological quality of studies included in the review was assessed independently by two reviewers (J.P.-H. and L.W.). A modified American National Institutes of Health (NIH): National Heart, Lung and Blood Institute Health Topics Study Quality Assessment Tool for ‘Before-After (Pre-Post) Studies With No Control Group’ was used.^40^ This approach to the risk of bias was chosen as many of the studies lacked a control, and similar reviews demonstrated its utility in higher education settings.^36^

The following outlines the 12 items from the tool used to determine the risk of bias: (item 1) clear study question; (item 2) prespecified eligibility criteria; (item 3) study participants representative; (item 4) all eligible participants enrolled; (item 5) sample size sufficiently large; (item 6) intervention clearly described and delivered consistently; (item 7) outcomes measures prespecified, valid, reliable and assessed consistently across all participants; (item 8) blinding; (item 9) 20% or less attrition in follow-up; (item 10) statistical methods examined changes in outcome measures/statistical tests conducted that provided *P*-values; (item 11) outcome measures taken multiple times before and after intervention; and (item 12) group level intervention took into account individual-level data to determine effects.^40^ For this review, items 8 and 12 were excluded, as they were irrelevant to any of the included studies.

For each study, all items were rated according to the guidance as ‘yes’ (met criteria), ‘no’ (did not satisfy criteria), ‘not reported’, ‘cannot determine’ (unclear from information) or ‘not applicable (not relevant to particular study).^40^ Reviewers used these ratings to make a qualitative assessment of overall risk of bias, using the ratings of ‘good’, ‘fair’ or ‘poor’. All risk-of-bias scorings are outlined for study in Supplementary Table 2.

### Effect measures

The baseline and post-intervention time points were used only in data extraction to calculate pre (time point 1) and post (time point 2) studies. The mean differences and *P*-values between pre and post of intervention and control group (when applicable) were calculated with raw data reported in individual longitudinal studies (if available). For cross-sectional studies with a control group, mean differences were calculated between groups at the post-intervention time point (as baseline data was not reported). Outcome data beyond post-intervention were not synthesised. When data was unavailable for calculating mean differences, ‘CD’ (cannot determine) was used.

Standardised mean differences (Cohen's *d*) with 95% confidence intervals were calculated when longitudinal studies included a control group. The calculations were made in StataMP version 17 for Windows,^41^ with the raw scores of each intervention/control measure, including sample size, mean difference and s.d. For longitudinal studies without a control group, available data such as *P*-value, Cohen's *d* and *t*-values were extracted. The significance of outcomes was also reported, which included the directionality of an improvement or decline.

### Synthesis methods

A meta-analysis was not appropriate because of the heterogeneity of study methodologies. The vote counting method outlined in the SWiM reporting guidelines was used.^33^ Missing data are denoted in the tables. Outcome data were tabulated for each included study and stratified by type of peer support intervention. The most common outcomes assessed in this review were stress, anxiety and depression. In each vote counting synthesis, the following was reported: the number and percentage of studies that affected the most common outcome for each peer support category, the binomial test indicating the probability of the results if the intervention was ineffective (i.e. equal to 0.5) and the 95% confidence intervals for the percentage of effects favouring the intervention.^42^ The binomial test was calculated in StataMP version 17,^41^ using the syntax ‘bitesti X Y 0.5’, whereas the 95% confidence intervals were calculated with the syntax ‘cii proportions X Y, level (95)’, where X equates to the number of effects and Y is the number of effects favouring the intervention.

## Results

### Study selection

**Fig. 1:**
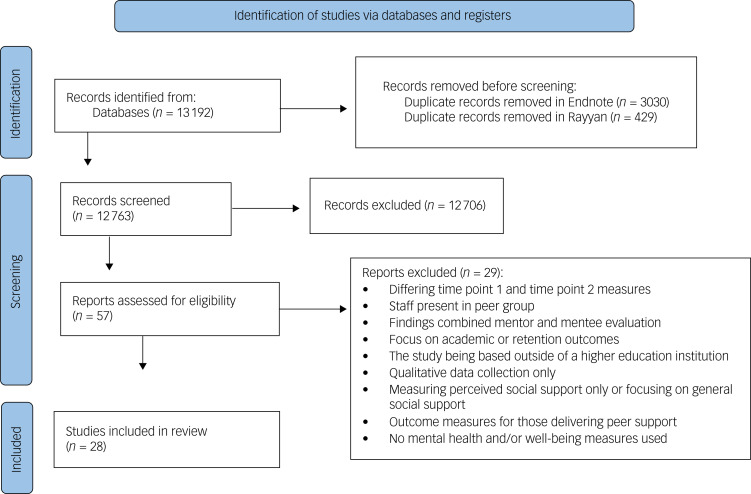
Process of identifying eligible studies for inclusion.

As summarised in Fig. 1, 12 763 records remained after duplicates were removed. A total of 57 papers were included for full-text screening, and a final 28 papers were included.

#### Study design characteristics

The study characteristics are outlined in Table 1 alphabetically according to the author, with a reference number used in square brackets for the results section only. The most common study type was the pre–post with a control design. Many studies (*n* = 12) adopted this approach [1, 4, 6–8, 14, 17, 20, 24–26, 28], whereas others (*n* = 7) employed a pre–post without controls design [2–3, 10–12, 15, 27]. Although some studies (*n* = 8) used a randomised controlled trial design [5, 9, 16, 18–19, 21–23], one of these studies [19] only used relevant mental health measures at time point 2, so that this study was analysed as a cross-sectional study with a control design along with one other study [13].

**Table 1:** Summary of study characteristics in review

[Reference number], (reference), country, type of HEI	Study design	Student population/mental health status of population	Intervention category	Student peer facilitator (reported training, year of study, course or characteristics)	Mode of delivery	Duration of intervention/number of sessions	Risk of bias
[1], (27), UK, university	Pre–post with control	First year	Peer learning: peer-assisted learning	Trained, senior	Face-to-face, group	1 semester, fortnightly	Fair
[2], (84), USA, university	Pre–post, no control	Nursing/over 30 years old with anxiety in clinical setting	Peer mentoring: peer dyad mentoring	Basic info for training, under age of 30, same clinical	Face-to-face, one-to-one	1 semester, 3 components	Good/fair
[3], (24), UK, university	Pre–post, no control	All	Peer-led support group: peer-run self-help groups	Trained	Face-to-face, group	6 sessions, weekly	Good
[4], (85), UK, university	Pre–post with control	First-year psychology	Peer mentoring	Trained, second and third year	Face-to-face, one-to-one	Academic year – no set meetings	Fair
[5], (86), USA, university	Randomised controlled trial	With mental illness	Peer-led support group: peer-led group-based intervention	Trained, mental illness	Face-to-face, group	5–6 weeks, with ≥3 booster sessions	Good
[6], (87), Turkey, technical university	Pre–post with control	Engineering	Peer learning: peer-led team learning	Second year, completed chemistry	Face-to-face, group	1 semester, 6 chemistry workshops	Fair
[7], (88), Turkey, university	Pre–post with control	All	Peer mentoring: peer-helping programme	Trained, counsellor candidates	Mixed: one-to-one and homework	Not reported, 15 activities	Good/fair
[8], (89), USA, university	Pre–post with control	Psychology	Peer-led support group: peer-led stress management group	Trained, male and female psychology students	Face-to-face, group	Not reported, 6 sessions	Good/fair
[9], (90), UK, university	Randomised controlled trial	With psychological problems	Peer-led support group: mutual support group	No facilitator – mutual support, with psychological problems	Online, group	10 weeks, not reported	Fair
[10], (91), USA, college	Pre–post, no control	Undergraduates	Peer-led support group: peer education/peer-facilitated stress management group	Peer educators	Face-to-face, group	Not reported, 2 sessions	Fair
[11], (92), USA, university	Pre–post, no control	First year	Peer mentoring	Trained, senior, paid	Face-to-face /video/telephone/chat, group (dyad)	3 weeks, 3 sessions	Poor
[12], (93), Australia, university	Pre–post, no control	First year, teacher education students during placement	Peer mentoring	Final year	Not reported, one-to-one	4 weeks, not reported	Poor
[13], (94), USA, university	Cross-sectional with control	Registered nurse anaesthetists	Peer mentoring	Second year	Not reported, one-to-one	2 years, not reported	Fair
[14], (95), USA, university	Pre–post with control	Senior social work	Peer-led support group: student-led support group	No facilitators – seniors took turns	Face-to-face, group	9 weeks, 9 sessions	Poor
[15], (96), USA, college	Pre–post, no control	Asian heritage/experience with race-related stress	Peer-led support group: peer-led group	Trained, Asian American	Face-to-face, group	8 weeks, 8 sessions	Fair
[16], (97), USA, college	Randomised controlled trial	All	Peer-led support group: group peer support led by peer leader	Trained, facilitated based on gender	Face-to-face, group	2 weeks, 2 sessions	Fair
[17], (98), Turkey, university	Pre–post with control	First and second years in psychology of learning and fundamentals of math	Peer learning: cooperative learning	No facilitators – took turns in course	Face-to-face, group	Not reported, once a week	Fair
[18], (99), USA, university	Randomised controlled trial	First year	Peer-led support group: peer-led social support group	Trained, clinical psychology honours of advanced course	Face-to-face, group	9 weeks, 8 sessions,	Good
[19], (100), USA, university	Randomised controlled trial (data used, cross-sectional w/control)	First year	Peer-led support group: peer-led social support group	Advanced clinical psychology with training guide	Face-to-face, group	9 weeks, 8 sessions	Fair/poor
[20], (101), USA, health professions college	Pre–post with control	Nursing enrolled in intro course	Peer mentoring	Oriented, second- or third-year nursing, completed intro course, clinical	Face-to-face, one-to-one	One semester	Fair
[21], (102), New Zealand, university	Randomised controlled trial	Second- and third-year medical	Peer-led support group: peer leaders	Trained, third-year medical	Face-to-face, group	25 weeks, approximately 21 sessions	Good
[22], (103), USA, university	Randomised controlled trial	Incoming first generation	Peer-led support group: group peer support	Trained, student therapists, clinical psychology with Master's and pursuing PhD	Face-to-face, group	7 weeks, 7 sessions	Good
[23], (104), USA, university	Randomised controlled trial	First-year athletes	Peer mentoring	Trained, already attended higher education	Face-to-face (<3 tele-mentoring sessions), one-to-one	16 weeks, meet 9 times	Good/fair
[24], (105), USA, university	Pre–post with control	At-risk Latino first-year students	Peer mentoring	Supervised, upper year/graduate counselling/psychology, paid	Face-to-face, one-to-one (1–3 mentees to 1 mentor)	Academic year, not reported	Poor
[25], (106), Australia, university	Pre–post with control	Second and third year paramedics	Peer-led support group: peer social support group	No facilitators – all trained in CARES (Connect to emotions, Attention training, Reflective listening, Empathy, Support help-seeking) skills for mutual support	Face-to-face, group	12 weeks, 6 sessions	Poor
[26], (107), UK, university	Pre–post with control	Third-year psychology	Peer-led support group: peer coaching	No facilitators – all trained in coaching psychology	Face-to-face, group	6 weeks, 5 sessions	Poor
[27], (108), Australia, university	Pre–post, no control	Undergraduates with autism who were mentees	Peer mentoring	Trained, postgraduate	Face-to-face, one-to-one	February to June, not reported	Fair
[28], (109), Canada, university	Pre–post with control	International	Peer mentoring	Canadian undergraduate	Face-to-face, one-to-one	One semester, encouraged to meet weekly	Fair

#### Population characteristics

Many studies (*n* = 13) targeted students by year of study, with the majority of studies offering peer support for lower-year students such as ‘first year’ [1, 4, 11–12, 18–19, 22–24] or ‘freshmen and sophomores’ [17]. Students were also recruited by discipline (*n* = 12); ‘nursing/nurse anaesthetists’ [2, 13, 20] and ‘psychology’ [4, 8, 26] courses were the most common. Other population criteria included ‘lived experience of mental health difficulties’ [5, 9], ‘student status’ [22, 28], ‘ethnicity’ [15, 24] and ‘age’ [2, 5, 20, 22]. The complete list is included in Table 1.

Other population characteristics were also extracted. One study focused on postgraduate students [13]. Others invited both undergraduates and postgraduates students to participate [1, 9]. All other studies were for undergraduate students. The majority of studies reported binary biological sex (male versus female). Of these, five reported the percentage of females in their sample only, leaving the reader to infer that the remaining percentage were males. Of the 22 studies that reported on binary sex in the baseline intervention group, the average proportion was 64.1% females and 35.9% males. Only one study used the term gender instead of sex in reporting; it was still presented in a binary way (44.4% men and 55.6% women [8]. Three studies [3, 13, 15] reported beyond binary sex, with options like ‘other’, ‘non-binary’ or ‘unspecified’ making up an average of 6.9%, along with 59.3% females and 33.8% males. The average mean age across the 20 studies that reported this for the intervention group was 21.6 years of age. Not enough studies reported clearly on gender, sex or mean age in the control group to desegregate this data. Similarly, few studies reported on ethnicity.

#### Intervention characteristics

Two intervention characteristics were important during this review: how a peer was defined and what type of peer support was investigated. To understand how the studies described a peer, we investigated how students were recruited for peer support (the population) and who facilitated the interventions. The studies referred to these students in various ways, including ‘leaders’, ‘peer supporters’ and ‘peer mentors’. This review uses the term ‘peer facilitators’ to describe any peer leading the intervention. Each study's population and peer facilitator are presented in Table 1. The shared experiences or identities between the peer facilitators and those accessing peer support helped to define a peer. Peer facilitators were frequently defined by their ‘seniority/year’ (*n* = 13) [1, 4, 6, 11–13, 19–24, 27] or ‘course of study’ (*n* = 11) [2, 6–8, 18–22, 24, 27]. A smaller number of studies recruited peer facilitators by ‘interest’ [3, 23], ‘gender’ [8, 16], ‘age’ [2], ‘lived experience’ of mental health difficulties’ [5, 9] or ‘heritage’ [15, 28]. Five studies created groups where all students participated and supported each other equally for mutual support [9, 14, 17, 25–26]. One study did not specify how they recruited [10]. These experiences and identities further defined being a peer beyond being a student in higher education.

The three categories of peer support created for this review to delineate between types are outlined below. A definition of each type is provided, along with the nomenclature process. The assigned category and each study's terminology (when different) are provided in Table 1.

##### Peer-led support group

This peer support gathers groups of students for mutual support. The most used terms of ‘peer-led/peer leader’ groups [5, 8, 14–16, 18, 19, 21] and ‘support groups’ [9, 14, 18–19, 25] or ‘group support’ [3, 16, 22] were both featured in eight studies.

##### Peer mentoring

Peer mentoring relies on higher-year/more experienced students to support lower-year/less experienced students. Eight studies used the term ‘peer mentoring’ [4, 11–13, 20, 23–24, 28], whereas two others used similar terms such as ‘specialised peer mentoring’ [27] or ‘peer dyad mentoring’ [2]. One study used ‘peer helper’ [7], but this was a one-to-one pairing of a more experienced student with a less experienced student.

##### Peer learning

This describes peer support that convenes students based on academic objectives. Terms used for this included ‘cooperative learning’ [17], ‘peer-assisted learning’ [1] and ‘peer-led team learning’ [6]. As the terms ‘peer’ and ‘learning’ were used across these studies, this category was named ‘peer learning’.

Most studies were categorised as a peer-led support group (*n* = 14) or peer mentoring (*n* = 11). The least common category of peer support was peer learning (*n* = 3).

The categorisation of these three types of peer support was most challenging with peer mentoring in small groups. Most peer mentoring occurred on a one-to-one basis; however, one study [24] paired mentors with one to three students. The potential small group, mutual nature of this type of peer support made a consideration of it being a peer-led support group necessary. Because the defining factor of this peer support study was that it was for incoming at-risk Latino students, its objective and ultimately self-identification as being a form of peer mentoring decided its final categorisation.

#### Comparator (control) characteristics

In total, 21 studies used a control group. Comparators in this review varied and included examples such as groups not participating in peer support [1, 6, 7, 13, 17, 20–22, 24, 25–26, 28], a waitlist [5, 8, 16], a group that met informally on occasion [18–19], a separate HEI without peer support [4], students given access to a website only [9], students in a different course or year (without peer support) [14] and faculty mentor pairing [23].

#### Outcome characteristics

There were 18 outcomes identified. Stress was most commonly measured with the Perceived Stress Scale^43^ (*n* = 8) [4, 10, 12, 14, 20, 22–24], with other measures being used only once, including the Chipas’ 2011 Survey^44^ [13] and the Depression, Anxiety and Stress Scale (DASS-21^45^) [25]. One study assessed stress by using two measures: the three-item House and Rizzo measure^46^ and Allen's^47^ two-item measure of mentor-related stress [11].

For anxiety, six measures were used: the State-Trait Anxiety Inventory (STAI)^48^ (*n* = 4) [1, 2, 6, 8], Generalised Anxiety Disorder-7 scale^49^ (*n* = 3) [1, 5, 21], Social Anxiety Questionnaire for Adults^50^ (*n* = 1) [6], Liebowitz Social Anxiety Scale^51^ (*n* = 1) [17], DASS-21^45^ (*n* = 1) [25] and the Adult Manifest Anxiety Scale – College Version^52^ (*n* = 1) [27].

Depression was assessed with the Beck Depression Inventory, Second Edition^53^ (*n* = 1) [15], Center for Epidemiologic Studies Short Depression Scale 10^54^ (*n* = 1) [5], DASS-21^45^ (*n* = 1) [25], ten-item Edinburgh Postnatal Depression Scale^55^ (*n* = 1) [24] and Patient Health Questionnaire-9^56^ (*n* = 1) [21].

Three studies measured well-being with the Shortened Warwick–Edinburgh Scale of Wellbeing^57,58^ (*n* = 1) [3], Positive and Negative Affect Schedule^59,60^ (PANAS; *n* = 1) [7] and Satisfaction with Life Scale^61,62^ (SWLS; *n* = 1) [7].

Loneliness was assessed with only one measure, the revised University of California – Los Angeles Loneliness Scale^63^ (*n* = 3) [17–19].

Psychological distress was measured with the Clinical Outcomes in Routine Evaluation – Outcome Measure^64^ (*n* = 1) [9], Brief Symptom Inventory^65^ (*n* = 1) [15] and the 12-item General Health Questionnaire^66^ (*n* = 1) [26].

The Index of General Affect from the Index of Wellbeing Scale^67^ (*n* = 1) [4] and the PANAS^68^ (*n* = 1) [16] measured negative affect.

These outcomes were measured in one study each: eating disorder pathology, measured with the Eating Disorder Examination Questionnaire^69^ [16]; resilience, measured with the 25-item Resilience Questionnaire^70^ [21]; quality of life, measured with the Linear Analogue Self-Assessment^71^ [21]; satisfaction with life, measured with the SWLS^72^ [9]; perceived social support, measured with the Social Provisions Scale^73^ [18]; domains of functioning, measured with the Outcomes Questionnaire^74^ [22]; belonging, measured with a 13-item questionnaire adapted for the study and based on the Institutional Integration Scale^75^ [24]; self-efficacy, measured with a 13-item adapted questionnaire^76,77^ [24]; and self-esteem, measured with Rosenberg's Self-Esteem Scale^78^ [4].

One study used multiple measurements for outcomes [28]. It explored psychological adaptation by using a six-item questionnaire similar to the PANAS^79^ and a four-item scale gauging life satisfaction.^80^ It also measured acculturative stress by using the homesickness and perceived discrimination subscales from the Acculturative Stress Scale for International Students,^81^ the language difficulty subscale from the Index of Life Stress^82^ and the Perceived Language Discrimination Scale^83^ [28].

### Quality assessment: risk of bias

The overall risk of bias for each study is outlined in Table 1. Out of the 28 included studies, five were rated ‘good’ and four were rated ‘good/fair’. In addition, 12 were rated as ‘fair’, one was rated as ‘fair/poor’ and six were rated as ‘poor’.

All studies stated their objective, clarified eligibility criteria, described the representativeness of the population, presented entry criteria, referred to the intervention and defined the well-being or mental health outcome. The quality ratings were thus determined according to sample size, attrition rate, statistical values and multiple time point measurement. Most (*n* = 22) studies were not adequately powered or did not report power analysis [1, 2, 4, 6–15, 17, 20, 22, 23–28]. Many (*n* = 13) had low retention, with loss to follow-up after baseline higher than 20% [3–4, 9, 13, 18–19, 25]. Other studies did not report enough information to determine attrition rates [1, 11, 14, 24, 26–27]. The statistical tests were not reported in five studies [12, 14, 19–20, 24]. Other studies did not report basic statistics such as the number of participants in the intervention/control group at pre- and post- time points, *P*-values, mean or s.d. at both baseline and follow-up [11–12, 14, 24]. Most studies (*n* = 20) had two time points and did not assess the outcome beyond the pre–post intervention [1, 2, 4, 6, 9–11, 13–15, 17, 19–22, 24–28].

If our synthesis was constrained to studies that were rated as ‘good’ or ‘good/fair’, we would retain studies. Of these, no peer learning would be represented. We only identified three studies of peer learning. All of these studies were rated as ‘fair’ with no power analysis reported and only two time points measured. Constraining the synthesis does not change the proportional representation of peer mentoring and peer-led support group studies.

### Individual study results

Every included study is outlined in Table 2, with the well-being and mental health outcome effect estimates provided where possible. A complete list of the acronyms and definitions of mental health and well-being measures are provided in Supplementary Table 3.

**Table 2:** Effect estimates for mental health/well-being outcomes per individual study

[Reference number], (reference), study design	Time points measured	Sample size Intervention group: time point 0/time point 1 Control group: time point 0/time point 1	Outcome	Mean difference in intervention group between baseline and follow-up (*P*-value)	Mean difference in control group between baseline and follow-up (*P*-value)	Results: standardised mean difference, 95% CI, Cohen's *d*, *t*-value, *W*-value, *P*-value	With control: favours intervention/favours control/no significant difference between groups No controls design: *s*ignificant improvement/decline – as reported by author(s)
Peer-led support group (*n* = 14) [3, 5, 8, 9, 10, 14–16, 18–19, 21–22, 25–26]							
[3], (24), Pre–post no control	3	Intervention group: 65/22	Mental well-being: SWEMWBS	1.77 (0.0103)*	Not applicable	*t*(35) = 2.98, *P* < 0.01, *d* = 0.56, 95% CI 0.17–0.94	Significant improvement
[5], (86), Randomised controlled trial	3	Intervention group: 63/49 Control group: 54/49	Depression: CES-D	−0.12 (0.31)	−0.22 (0.10)	SMD = 0.17, 95% CI −0.20 to 0.54	Not significant
			Anxiety: GAD-7	0.00 (1.00)	0.69 (<0.0001)*	SMD = −0.91, 95% CI −1.30 to −0.51	Not significant
[8], (89), Pre–post with control	3	Intervention group: 18/15 Control group: 18/12	State anxiety: STAI	−0.62 (0.87)	5.85 (0.26)	SMD = −0.53, 95% CI −1.2 to 0.14	Favours intervention
[9], (90), Randomised controlled trial	2	Intervention group: 142/51 Control group: 141/82	Psychological distress: CORE-OM,	−0.22 (0.02)*	−0.27 (0.0027)*	SMD = 0.08 95% CI −0.15 to 0.31	Not significant
			SWLS	1.20 (0.31)	2.66 (0.0086)*	SMD = −0.20, 95% CI −0.43 to 0.03	Not significant
			Sense of Community Index	0.05 (0.42)	0.00 (1.00)	SMD = 0.14, 95% CI −0.09 to 0.37	Not significant
[10], (91), Pre–post no control	2	Intervention group: 48/48	Stress: PSS	Study skills intervention group: −3.86 (<0.001)* Mindfulness intervention group: −1.96 (<0.05)*	Not applicable	*t*(46) = −1.47, *P* = 0.15	Significant improvement
[14], (95), Pre–post with control	2	Intervention group: 14/13 Control group: 13/13	Stress: PSS	Cannot determine	Cannot determine	Cannot determine	Not significant – statistics not reported, only statement
[15], (96), Pre–post no control	2	Intervention group: 10/10	Depression: BDI-II	−6.00 (0.11)	Not applicable	BDI-II: *t*(9) = 2.03, *P* < 0.07, *d* = 0.76	Significant decline
			Psychological distress: BSI	−0.43 (0.0431)*	Not applicable	BSI: *t*(9) = 2.91, *P* < 0.05, *d* = 0.99	Significant decline
[16], (97), Randomised controlled trial	4	Intervention group: 126/112 (female FO: 41/39, female MG: 49/39, male MG: 36/34) Control group: 70/56 (female: 36/33, male: 34/23)	Negative affect: PANAS	Cannot determine	Cannot determine	Cannot determine	Not significant
			Eating disorder pathology: EDE-Q	Cannot determine	Cannot determine	Cannot determine	Not significant
[18], (99), Randomised controlled trial	3	Intervention group: 82/65 Control group: 73/47	Loneliness: UCLA	−0.12 (0.10)	0.12 (0.22)	SMD = −0.50, 95% CI −0.82 to −0.18	Favours intervention
			Perceived social support: SPS	0.10 (0.07)	−0.02 (0.81)	SMD = 0.29, 95% CI −0.03 to 0.60	Favours intervention: (if attended more than 3 sessions)
[19], (100), Time point 2 cross-sectional with control	1	Intervention group: 88/51 Control group: 82/45	Loneliness: UCLA	0.20 (<0.0001)*		*r*(94) = –0.25, *P* < 0.05	Favours intervention
[21], (102), Randomised controlled trial	2	Intervention group: 133/111 Control group: 142/121	Depression: PHQ-9	−0.46 (0.35)	0.20 (0.64)	SMD = −0.18, 95% CI −0.41 to 0.06	Not significant
			Anxiety: GAD-7	−0.23 (0.61)	−0.06 (0.89)	SMD = −0.05, 95% CI −0.29 to 0.19	Not significant
			Quality of life: LASA	0.06 (0.75)	−0.14 (0.48)	SMD = 0.13, 95% CI −0.11 to 0.36	Not significant
			Resilience: RS15	1.47 (0.37)	0.60 (0.71)	SMD = 0.07. 95% CI −0.17 to 0.31	Not significant
[22], (103), Randomised controlled trial	2	Intervention group: 25/21 Control group: 31/31	Domains of functioning: OQ-45.2	−11.81 (0.07)	−3.16 (0.56)	SMD = −0.43, 95% CI −0.96 to 0.10	Favours intervention
			Stress: PSS-10	−3.14 (0.16)	−2.23 (0.14)	SMD = −0.14, 95% CI −0.66 to 0.39	Not significant
[25], (106), Pre–post with control	2	Intervention group: 76/51 Control group: 52/37	Depression, anxiety, stress: DASS-21	4.00 (0.0164)*	7.00 (0.0001)*	SMD = −0.37, 95% CI −0.72 to −0.01	Not significant
[26], (107), Pre–post with control	2	Intervention group: 32/not reported Control group: 33/not reported	Psychological distress: GHQ-12	0.37 (not significant)	5.07 (0.002)*	SMD = −0.56, 95% CI −1.06 to −0.07	Not significant
Peer mentoring [2, 4, 7, 11–13, 20, 23–24, 27–28]							
[2], (84), Pre–post no control	2	Intervention group: 16/16	Anxiety: STAI	−7.75 (0.0193)*	Not applicable	Pre-test 95% CI 34.59–46.03, post-test 95% CI 29.12–36.00	Significant decline: anxiety
[4], (85), Pre–post with control	2	Intervention group: 117/56 Control group: 112/53	Self-esteem: Rosenberg's Self-Esteem Scale;	0.29 (0.73)	−1.08 (0.16)	SMD = 0.27, 95% CI 0.01–0.53	Not significant
			Negative affect: Index of General Affect (from Index of Wellbeing);	−2.39 (0.0560)*	1.350 (0.37)	SMD = −0.45, 95% CI −0.71 to −0.19	Favours intervention
			Stress: PSS (time point 2)	Time point 2 stress: 1.26 (0.26), cross-sectional with control		Cannot determine	Not significant
[7], (88), Pre–post with control	2	Intervention group: 30/26 Control group: 30/26	Well-being: PANAS positive affect	6.77 (0.0024)*	−2.77 (0.10)	SMD 1.36, 95% CI 0.80–1.93	Favours intervention
			negative affect	−8.11 (0.0002)*	−1.45 (0.41)	SMD = −0.82, 95% CI −1.35 to −0.29	Favours intervention
			SWLS	6.43 (<0.0001)*	1.60 (0.21)	SMD = 1.03, 95% CI 0.49–1.57	Favours intervention
[11], (92), Pre–post no control	2	Intervention group: 271/not reported	School-related stress: 3-item Anxiety-Stress Questionnaire, mentor-related stress reduction: 2-item measure	Cannot determine	Not applicable	*t* = 1.79, *P* = 0.04, two-tailed	Significant improvement (for those who received greater psychosocial support from mentor according to adapted 25-item)^110^
[12], (93), Pre–post no control	3	Intervention group: 2/2	Stress: PSS	Cannot determine	Not applicable	Cannot determine	Significant improvement
[13], (94), Time point 2 cross-sectional with control	1	Intervention group: 26 Control group: 11	Stress: Chipas’ 2011 Survey	1.27 (0.02)*		*t*(35) = 2.35, *P* = 0.025	Favours intervention
[20], (101), Pre–post with control	2	Intervention group: 25/23 Control group: 26/24	Stress: PSS	−0.53 (0.75)	0.75 (0.66)	SMD = −0.21, 95% CI −0.76 to 0.34	Not significant
[23], (104), Randomised controlled trial	2	Intervention group: 31/28 Control group: 30/29	Stress: PSS	1.96 (0.25)	2.48 (0.11)	SMD = −0.09, 95% CI −0.60 to 0.41	Not significant
[24], (105), Pre–post with control	2	Intervention group: 34/not reported Control group: 37/not reported	Self-efficacy: Adaptation of ^76,77^	Efficacy, 0.2 (cannot determine)	Efficacy, 0.02 (cannot determine)	Efficacy *t* = 2.58, d.f. = 24, *P* = 0.015 (intervention group only)	Not significant
			Belonging: Institutional Integration Scale,	0.28 (cannot determine)	−0.01 (cannot determine)	*t* = 2.34, d.f. = 24, *P* = 0.03 (intervention group only)	Not significant
			Depression: EPDS	−0.64 (cannot determine)	0.18 (cannot determine)	F(1, 41) = 5.51, *P* = 0.029	Favours intervention
			Stress: shortened PSS	−0.26 (cannot determine)	0.06 (cannot determine)	F(1, 71) = 4.36, *P* = 0.04	Favours intervention
[27], (108), Pre–post no control	2	Intervention group: 10/10	Anxiety: AMAS-C	−2.60 (0.08)	Not applicable	*d* = 0.58 *P* = 0.08	Not significant
[28], (109), Pre–post with control	2	Intervention group: 23/25 Control group: 22 (time point 2)	Acculturative stress: ASSIS, Language difficulty (from Index of Life Stress), Perceived Language Discrimination Scale	−0.31 (0.21)	−0.48 (0.04) (cross-sectional intervention group to control group at time point 2)	*t*(22) = 2.9, *P* = <0.001, *d* = 0.64, 95% CI 0.10–0.58 (pre–post intervention group) *t*(45) = −2.1, *P* = <0.05, *d* = 0.61, 95% CI −0.94 to −0.02 (time point 2 intervention group to control group)	Significant improvement (pre–post intervention group) Favours intervention (cross-sectional with control at time point 2)
			Psychological adaptation: 4-item scale of life satisfaction and 6-item scale similar to the PANAS	0.31 (0.07)	0.46 (0.04) (cross-sectional intervention group to control group at time point 2)	t(22) = −1.89, *P* < 0.05, *d* = 0.57, 95% CI −0.61 to 0.03 (pre–post intervention group) *t*(45) = 2.08, *P* = <0.05, *d* = 0.60, 95% CI 0.01–0.92 (time point 2 intervention group to control group)	Significant improvement (pre–post intervention group) Favours intervention (cross-sectional with control at time point 2)
Peer-led learning [1, 6, 17]							
[1], (27), Pre–post with control	2	Intervention group: 29/29 Control group: 54/54	Anxiety: GAD-7 and STAI	4.14 (0.10)	8.52 (0.0002)*	SMD = −0.40, 95% CI −0.86 to 0.05	Favours intervention
[6], (87), Pre–post with control	2	Intervention group: 68/68 Control group: 60/60	State anxiety: STAI-S	−7.31 (0.0001)*	−3.01 (0.08)	SMD = −0.60, 95% CI −0.95 to −0.24, Cohen's *d* = 0.55	Favours intervention
			Social anxiety: SAQ	−10.35 (0.0007)*	−10.30 (0.0020)*	SMD = −0.00, 95% CI −0.35 to 0.34, Cohen's *d* = 0.68	Not significant
[17], (98), Pre–post with control	2	Intervention group: 57/57 Control group: 57/57	Loneliness: R-UCLA	−4.53 (0.0055)*	−0.05 (0.98)	SMD = −0.511, 95% CI −0.89 to −0.14	Favours intervention
			Social anxiety: LSAS	−10.73 (0.0103)*	0.89 (0.78)	SMD = −0.79, 95% CI −1.17 to −0.41	Favours intervention

Note: Information not reported within the table was not reported in the reviewed studies.

SWEMWBS, Shortened Warwick–Edinburgh Scale of Wellbeing; CES-D, Center for Epidemiologic Studies Depression Scale; GAD-7, Generalised Anxiety Disorder; SMD, Standardized Mean Difference; STAI, State-Trait Anxiety Inventory; CORE-OM, CORE Outcome Measure; SWLS, Satisfaction with Life Scale; PSS, Perceived Stress Scale; BDI-II, Beck Depression Inventory; BSI, Brief Symptom Inventory; PANAS, Positive and Negative Affect Schedule; EDE-Q, Eating Disorder Examination Questionnaire; UCLA, University of California – Los Angeles; SPS, Social Provisions Scale; PHQ-9, Primary Health Questionnaire; LASA, Linear Analogue Self-Assessment; RS15, Resilience; OQ-45.2, Outcomes Questionnaire; DASS-21, Depression, Anxiety and Stress Scale; GHQ-12, General Health Questionnaire; EPDS, Edinburgh Postnatal Examination Questionnaire; AMAS-C, Adult Manifest Anxiety Scale – College Version; ASSIS, Acculturative Stress Scale for International Students; SAQ, Social Anxiety Questionnaire for Adult; R-UCLA, Revised University of California – Los Angeles; LSAS, Liebowitz Social Anxiety Scale.

* *P* < 0.05.

### Results of syntheses

The most frequent outcomes evaluated were stress, anxiety and depression (for peer-led support groups only). Vote counting is reported for these outcomes based on the direction of effect with the binomial probability test and 95% confidence intervals. Effect estimates for less frequently reported outcomes with sufficient data available are reported in Table 2.

### Peer-led support group

Four studies analysed the effect of the intervention on depression. One study had significant results (25%, 95% CI 0.63–80.59%, *P* = 0.625) with a decline in depression symptoms; however, its risk of bias was ‘fair’. The other three studies all found no significant results.

Three studies reported the effect of the intervention on anxiety. One study (33.3%, 95% CI 0.84–90.57%, *P* = 1.00) favoured the intervention with reduced anxiety and was rated as ‘good/fair’ in the risk-of-bias assessment. Two studies found no significant results for anxiety.

Three studies analysed stress as an outcome. One study (33.3%, 95% CI 0.84–90.57%, *P* = 1.00) had a significant decline in stress, but it was rated as ‘fair’ in the risk-of-bias assessment. One study in this category did not have any significant findings for stress, whereas the other had mixed results, with no significant findings for stress but significant improvements in functioning.

### Peer mentoring

Two peer mentoring studies measured anxiety. One had a significant decrease in anxiety; the other found non-significant results.

Five studies (62.5%, 95% CI 24.49–91.48%, *P* = 0.73) found significant results for stress. Of these significant positive results for stress, two of the studies were rated as ‘fair’ following risk-of-bias assessment. The other studies were rated as ‘poor’. Three studies found no significant reduction in stress, but one of these had mixed results, with significant improvements to negative affect.

### Peer learning

All three peer learning studies measured anxiety. Although one study had mixed results, the other two reported significant intervention effects (66.67%, 95% CI 9.43–99.16%, *P* = 1.00). All three studies were rated as ‘fair’ in their risk-of-bias assessment, with no power analysis reported and only two time points measured.

## Discussion

This review demonstrates a wide variation in interventions and terminology used to describe peer support. Although many use the label to encapsulate all forms of peer support, this does not capture the nuances of different peer support interventions. Previous reviews only using peer support as a search term exhibit this, finding just three studies and missing relevant work.^21^ We found peer support for student mental health and well-being referred to as everything from cooperative learning to peer-led social support groups. There is little consistency in the terminology. Without a shared vocabulary, it is difficult to understand how different forms of peer support may benefit higher education students. This review identified three main categories of peer support: peer-led support groups, peer mentoring and peer learning. A shared understanding and use of these categorical terms beyond peer support is imperative to future research and dissemination. However, first the definition of a peer needs to be clarified.

### Defining a peer

The lack of consistent terminology brings into question how HEIs define a peer. Although peer support is broadly about people supporting each other based on shared experiences,^12^ more is required to define a peer in HEIs. This review defined peer support as higher education students helping each other since all peer facilitators and students accessing peer support had this identity. However, other identities are also being used to define a peer by ‘course’, ‘year/seniority’, ‘heritage’, ‘age’ and ‘lived experience of mental health difficulties’.

Of the studies that defined peers based on their year of study (*n* = 13), ten were for first-year students. Although this may not be surprising for peer mentoring, as it is defined by a higher-year student supporting a lower-year student, this was also seen in peer-led support groups and peer learning. Being described as an ‘acute stressor’, the transition into higher education strains well-being, as students face many changes and can struggle to settle in.^111^ Perhaps this is why so many peer support interventions are focused on first-year students; however, each year in higher education presents new challenges, with stress levels fluctuating throughout a degree. Conley et al^112^ found that students in the USA enrolled in a 4-year degree had the poorest psychological functioning across the first two years of study, with improvements seen in the final two years. In England, anxiety triggered by higher education and psychological well-being fluctuated for 3-year degree students; however, depression rates were highest in the final year of study.^113^ This finding raises questions about whether students would also benefit from peer support beyond their first year. Of the four papers that offered peer support for higher-year students such as those in their second and third years,^102,106^ third years^107^ and seniors,^95^ all were part of the peer-led support group category. None had significant results for improved student mental health and well-being outcomes. Although a need might exist, more research is needed to understand if peer support does improve the mental health and well-being of higher-year students.

Another common way to define a peer was through a course of study. Healthcare studies and psychology were the most frequent courses to offer peer support, with nine of the 12 studies falling within these disciplines. Compared with students from other degrees, studies indicate that medical students have higher rates of mental and emotional difficulties, increased levels of mental distress during training and are less likely to seek help.^114,115^ In one study, however, students from the sciences and arts and humanities had significantly higher mean levels of depression than students from health sciences and social sciences.^116^ A study of nursing students in Spain and Chile found that levels of mental distress reduced over time, indicating that nursing education may be a protective factor against mental health disorders.^117^ Therefore, peer support should be evaluated with students across various courses to understand any differences.

### Peer-led support groups

This review defined peer-led support groups as a type of peer support that aims to gather groups of students together for mutual support, which was a unique factor. Mutual support is ‘a process by which persons voluntarily come together to help each other address common problems or shared concerns’.^118^ Peers form self-help support groups by meeting for mutual assistance.^24^ Although group settings offer mutual support for those attending, the review did not include outcomes for peer facilitators, so the mutuality of these groups warrants further investigation. From descriptions alone, it is hard to discern the extent of mutuality in support provision. Of the four studies that had all students act as facilitators,^95,98,106,107^ all were part of the peer-led support group category except one, which was categorised as peer learning.^98^ One study had facilitators take turns leading,^87^ whereas the others had all students trained with no set facilitator for the group sessions so that everyone was expected to participate equally.^106,107^

Peer-led support groups had the most mixed findings, so their efficacy remains to be seen. As the most frequently evaluated intervention type, 20 measures were used to explore 14 mental health and well-being outcomes. The various measures might demonstrate indecision on the objective of peer-led support groups. Similarly, the different measures could also be explained by the different delivery methods. Although the ‘group’ aspect of this category was the defining feature, the studies represented a range of interventions, such as a peer-run self-help group,^24^ mutual support group^90^ and peer-facilitated/-led stress management group/peer education.^89,91^ The diverse delivery methods may explain the difference in outcome measures assessed and the mixed results of this category. However, many studies lacked detailed descriptions of the interventions. Hence, it is difficult to assess whether they are indeed distinct or if a difference in the nomenclature used to describe interventions explains these results. Based on the heterogenous literature for this peer support, it is impossible to identify when or why some forms improve student mental health. The peer-led support group is therefore a category of peer support that warrants further investigation using shared terminology and clear descriptions of the interventions to understand the factors associated with its efficacy.

### Peer mentoring

In this review, we defined peer mentoring as a type of peer support that relies on higher-year/more experienced students to support lower-year/less experienced students. Mentoring is known broadly as a transfer of knowledge,^119^ where a more experienced, usually older, individual guides a mentee with less experience.^120^ Depending on the institution, peer mentoring goes by names such as a ‘parent’ programme, ‘buddy’ scheme or ‘family’ programme. No matter the title, peer mentoring programmes operate on the same belief that students who have more experience in higher education can mentor less experienced students.

Peer-led support groups were defined by their group nature; peer mentoring had more heterogeneity in approach. Most peer mentoring happened with mentors supporting mentees on a one-to-one basis, but three of the 11 papers took alternative approaches. These studies paired one mentor with up to three mentees,^105^ had a dyad with a group of mentees connecting with one mentor^92^ or took a mixed approach with one-to-one meetings and homework assigned to the students receiving support.^88^ All alternative approaches to one-to-one peer mentoring had significant results in the assessed mental health and well-being measures. Overall, the included studies used 17 measures to evaluate ten outcomes. Stress was the predominant outcome, with 62.5% of the studies demonstrating significant, positive results for stress. Therefore, peer mentoring benefits student stress and takes a mostly one-to-one structure; however, other approaches can be helpful. The literature mostly agrees on peer mentoring terminology to describe this type of peer support.

### Peer learning

This review defined peer learning as a type of peer support that convenes students based on academic objectives and tends to be situated in departments. Peer-led team learning^87^ and cooperative learning^98^ contributed to this category. Cooperative learning creates spaces where students work toward a common purpose and assist each other in learning.^121,122^ Peer-led team learning is an experiential learning environment where students build knowledge, talk to each other and develop higher-level reasoning and problem-solving skills by thinking together about the conceptual side of learning.^123–125^ Peer-assisted learning is also part of this category,^27^ with Bournemouth University defining it as ‘a scheme that fosters cross-year support between students on the same course’ while encouraging students to learn together and help each other.^126^ The approach to learning is socially focused.^127^ In this way, peer learning is distinguished from other supportive activities because it is facilitative of student learning; structured and purposeful with training and support; reliant on small groups; open to everyone, non-compulsory and takes place in a safe, more relaxed environment.^128^

Peer learning traditionally focuses on academic objectives. As such, there are few studies assessing the impact of this type of peer support on mental health and well-being. The data captured here, however, suggests that peer-led learning interventions may improve student mental health, with a significant impact on reducing anxiety. Thus, the positionality of peer learning in departments may be an opportunity for HEIs to take a settings-based approach to improve student mental health in the classroom.

### Peer support in higher education versus community peer support

Although the promise of peer support in higher education is underpinned by the more established body of research on peer support in community health settings, two issues have been raised through this review. First, the measures being used differ. Two meta-analyses found significant reductions in depressive symptoms for peer support as an intervention in communities,^20,129^ which have been used to justify further exploration of peer support in higher education. Depression was measured as an outcome in only five studies in the higher education context. Of these, one was peer mentoring, which significantly favoured the intervention. The others were peer-led support groups, with only one of the four studies reporting significant benefits to depression. The lack of depression measures makes comparing findings in community settings to HEIs difficult.

Only two peer-led support group studies defined a peer based on their lived experience of mental health difficulties,^86,90^ bringing them together with peer facilitators who self-identified as living with a ‘mental illness’ or ‘psychological problem’. This finding contrasts the definitions of a peer used in community mental health settings. The NHS website defines peer support workers as ‘people who have lived experience of mental health challenges themselves’ and who use their experiences to empathise with and support others. This inconsistency in how HEIs classify a peer in contrast to how a peer is defined in community mental health settings in the UK is essential. Because peer support in higher education does not seem to recruit facilitators or students based on lived experience with mental health difficulties, the basic definitions of a peer in a community versus a HEI differ. This disparity in definition and lack of shared outcome measures mean that the comparison between community programmes and peer support in higher education cannot currently be made with the literature.

### Limitations of evidence included in the review

No grey literature met the inclusion criteria. A search was undertaken in OpenGrey^34^ and Grey Matters,^35^ but no results were found. In addition, no relevant grey literature was encountered through cross-referencing the included full-text studies. Although five reports were discovered in a scoping review, all were excluded after screening. They reported on peer support in higher education generally, undertook qualitative evaluation only or did not use a measure of student mental health or well-being that fit the study criteria.^12,30,130–132^ Although grey literature can reduce publication bias and improve the comprehensiveness of a systematic review,^133^ more robust reporting in grey literature is needed to meet basic efficacy measures in higher education peer support.

Most included studies lacked a power analysis to assess whether sample sizes were sufficient to detect intervention effects. Of those that reported a power analysis, many had poor retention and/or small sample sizes, which may explain the many non-significant results of this review. Of the 28 included studies, 21 did not report a power analysis. One included study was a primarily qualitative study, where the quantitative element met the inclusion criteria, but the sample size was small (*n* = 2), affecting its quality.^93^ Of the seven studies that did report a power analysis, one did not achieve the sample size required.^94^ Four of these were rated as ‘good’ in the risk-of-bias assessment, but the two others were rated ‘fair’^97^ and ‘fair/poor’^100^ because of low retention and poor reporting of outcome measures. A similar review in higher education settings also found many underpowered studies, indicating the need to run interventions to broader cohorts of students across faculties, programmes or similar institutions to improve power.^36^ With only six studies reporting on the funding received, more funding may be required to make adequately powered studies a reality.

Many studies presented incomplete data; for example, unclear sample sizes and missing statistics/ raw data (i.e. means and s.d.). Demographics were also poorly reported, so that it was not possible to disaggregate gender, age or ethnicity for a helpful discussion. Despite many studies missing integral parts, available data were extracted when possible to calculate mean differences, *P*-values and standardised mean differences for a more consistent synthesis. The reporting in this review may indicate that better guidelines are required. One review of higher education interventions for student mental health and well-being recommended that medical reporting guidelines^134,135^ are adapted to improve standards.^36^

Outcome measures were too heterogenous for meaningful comparison. Although anxiety and stress were the most common outcomes investigated in the literature, there was little consistency in measures. Although the Perceived Stress Scale was used most to measure stress (*n* = 8) and the STAI was used to measure anxiety (*n* = 4), many other measures were also applied to assess these common outcomes. Some measures, such as the PANAS, were used to measure different outcomes. For example, Eryilmaz^88^ chose to use PANAS and the SWLS to measure subjective well-being, Kilpela et al^97^ used PANAS to measure negative affect and Thomson and Esses^109^ used PANAS to measure psychological adaptation. This lack of consistency is an obstacle to comparing and drawing conclusions on effective interventions. A ‘core set’ of well-being measures validated in higher education student populations has been recommended.^136^ Similar guidance is needed for stress, anxiety and perhaps depression, as this review's most common outcome measures, to complement existing toolkits.^137^

### Limitations of the review process

A meta-analysis was not possible because of the outcome measure heterogeneity, few reported effect sizes (or raw data to calculate them) and limited information on the interventions to compare similar studies. Vote counting is considered a less robust way to synthesise evidence in a systematic review, since no information is given on the magnitude of effects, sample sizes are not considered and combining *P*-values is a more robust method.^138^ This systematic review is limited by the narrative synthesis taken; however, using SWiM guidelines improved reporting transparency.^33^ Nonetheless, the synthesis method stipulated by the current evidence available in the field limits the conclusions that can be drawn.

Although the Cochrane tool for assessing risk of bias in randomised trials and other such tools is widely used,^139^ most do not support multiple study designs.^36^ As this review had seven randomised controlled trials, two cross-sectional with control and 19 pre–post with and without control designs, a different tool was required. A modified NIH ‘Quality Assessment Tool for ‘Before-After (Pre-Post) Studies With No Control Group’ was used for the risk-of-bias assessment.^40^ The chosen method was limited in practice because it is designed for studies without a control group, so there were no criteria acknowledging if a study had a control group, which would strengthen its quality. This approach to risk-of-bias assessment was best-suited for the heterogeneity of our included studies;^36^ however, as some studies also had a cross-sectional design with a comparator, the chosen tool was an imperfect option.

In this study's synthesis, the initial baseline and post-intervention measures were included for pre–post intervention outcome measures. The post-intervention measures were synthesised for cross-sectional with a control design. This approach was used because studies included a mix of interim and follow-up measures at varying durations that did not allow for comparison. Although all time points were extracted to see if comparable data was available, only the pre–post measures for longitudinal studies and cross-sectional post-intervention data with control could be used for synthesis. Using the pre–post time points allowed for more comparison and generalisability in the extraction and synthesis process.

Finally, the methodology has an additional limitation. This paper focused on quantitative studies to meet the second of our objectives: to evaluate the effectiveness peer support in higher education. Future work may benefit from reviewing qualitative studies to confirm our categorisation of types of peer support and definitions of peer.

### Implications of the results for practice, policy and future research

This systematic review found that peer support in higher education is defined in the literature according to three categories: peer-led support groups, peer mentoring and peer learning. By identifying this nomenclature, HEIs can start using a shared language when evaluating interventions and communicating best practice. It will also improve understanding of the strengths and limitations of peer support in more detail so that areas for further research can be prioritised.

Peer-led support groups come together for mutual support. Exploring the mutuality of peer support for the peer facilitators and those attending was beyond the scope of this review, but should be studied further. In addition, although this form of peer support was the only one to measure depression outcomes multiple times, results were mixed, which may indicate that the category is too broad. Alternatively, as this form of peer support is most comparable to community mental health settings, it may be that the gap in how HEIs and healthcare settings define a peer and measure different outcomes is the barrier to the identification of effective interventions. Further investigation is needed into what specific peer-led support group components improve efficacy.

Peer mentoring is mostly for incoming students to receive support from a higher-year/more experienced student. This type of peer support was the most homogeneous in the terminology used and implementation (one to one). Of the three peer support types, it was also the most promising for improving stress outcomes. Nonetheless, alternative approaches to peer mentoring (e.g. small groups) demonstrated significant results in other measures (e.g. affect and depression), indicating that more research is needed to understand how the structure of peer mentoring affects mental health and well-being outcomes.

Peer learning operates in groups and convenes for academic objectives. Results indicate that significant improvements in anxiety were linked to peer learning. HEIs should consider incorporating relevant measures into existing peer learning programmes so that further investigations of its benefits to mental health and academic outcomes can be made.

In conclusion, despite hopes that peer support in higher education would offer an accessible, setting-based solution to improving student support, the findings of this review are mixed. Of the three types of peer support, two had the most significantly positive results: peer learning reduced anxiety and peer mentoring reduced stress levels. Results for peer-led support groups, however, were varied. Although peer-led support group interventions assessed depression more than any other type of peer support, they did not show a majority of significant results for any of the outcomes measured.

Peer support interventions aimed at improving student mental health and well-being were set up with specific objectives, such as easing the transition into higher education (peer mentoring), meeting academic objectives (peer learning) or enhancing mutual support (peer-led support groups). Furthermore, how a peer was defined in the higher education context varied, which is crucial to understanding the intervention. Students’ years of study and discipline were common features of defining a peer. However, peer-led support groups were the only type that brought together students with lived experiences of mental health difficulties as peers, which is most similar to community mental health settings. This comparability warrants further investigation, as this type of peer support shows promising applications in wider communities.

Various modes of peer support that use specific definitions of a peer are more or less useful for different needs. Although HEIs consider peer support as a potential addition to support services, defining the type of peer support and what a peer is must be considered. Next, researchers and educators need to set standardised mental health and well-being metrics for the various types of peer support, so that more robust studies can be conducted. These findings should be shared widely, using better reporting guidance to elevate best practice. With this, HEIs can start to assess which types of peer support are helpful when and for whom, as part of a whole-university approach to support all students’ mental health and well-being. The definitions of peer support provided in this review, however, are the first steps toward a consistently shared vocabulary to tackle these challenges.

## Supporting information

Pointon-Haas et al. supplementary material Pointon-Haas et al. supplementary material

## Data Availability

The data that support the findings of this study are available from the corresponding author, J.P.-H., upon reasonable request.

## References

[1] Brown JSL. Student mental health: some answers and more questions. J Ment Health 2018; 27(3): 193–6. 29768071 10.1080/09638237.2018.1470319

[2] Neves J, Hillman N. Student Academic Experience Survey 2017. Higher Education Academy and Higher Education Policy Institute, 2017 (https://eric.ed.gov/?id=ED603568).

[3] National Careers Service. *Higher Education*. National Careers Service, 2023 (https://nationalcareers.service.gov.uk/explore-your-education-and-training-choices/higher-education).

[4] Auerbach RP, Mortier P, Bruffaerts R, Alonso J, Benjet C, Cuijpers P, et al. WHO world mental health surveys international college student project: prevalence and distribution of mental disorders. J Abnorm Psychol 2018; 127(7): 326–68. 30211576 10.1037/abn0000362 PMC6193834

[5] Thorley C. Not by Degrees: Improving Student Mental Health in the UK's Universities. Institute for Public Policy Research, 2017 (https://www.ippr.org/publications/not-by-degrees).

[6] Higher Education Statistics Agency (HESA). *Table 15 UK Domiciled Student Enrolments by Disability and Sex 2014/15 to 2019/20*. Higher Education Statistics Agency, 2020 (https://www.hesa.ac.uk/data-and-analysis/students/table-15).

[7] Whitelaw S, Baxendale A, Bryce C, MacHardy L, Young I, Witney E. ‘Settings’ based health promotion: a review. Health Promot Int 2001; 16(4): 339–53. 11733453 10.1093/heapro/16.4.339

[8] Fernandez A, Howse E, Rubio-Valera M, Thorncraft K, Noone J, Luu X, et al. Setting-based interventions to promote mental health at the university: a systematic review. Int J Public Health 2016; 61(7): 797–807. 27364779 10.1007/s00038-016-0846-4

[9] Dooris M, Poland B, Kolbe L, de Leeuw E, McCall DS, Wharf-Higgins J. Healthy settings. In Global Perspectives on Health Promotion Effectiveness (eds DV McQueen, CM Jones): 327–52. Springer, 2007.

[10] Okanagan Charter: An International Charter for Health Promoting Universities & Colleges. International Conference on Health Promoting Universities & Colleges / VII International Congress (University of British Columbia, 22–25 Jun 2015). University of British Columbia, 2016 (https://open.library.ubc.ca/cIRcle/collections/53926/items/1.0132754).

[11] Hughes G, Spanner L. The University Mental Health Charter. Student Minds, 2019 (https://www.studentminds.org.uk/uploads/3/7/8/4/3784584/191208_umhc_artwork.pdf).

[12] Gulliver E, Byrom N. Peer Support For Student Mental Health. Student Minds, 2014 (https://kclpure.kcl.ac.uk/portal/en/publications/peer-support-for-student-mental-health-a-review-of-the-use-of-pee).

[13] Solomon P. Peer support/peer provided services underlying processes, benefits, and critical ingredients. Psychiatr Rehabil J 2004; 27(4): 392–401. 15222150 10.2975/27.2004.392.401

[14] Mead S, Hilton D, Curtis L. Peer support: a theoretical perspective. Psychiatr Rehabil J 2001; 25(2): 134–41. 10.1037/h0095032 11769979

[15] Schubert MA, Borkman T. Identifying the experiential knowledge developed within a self-help group. In Understanding the Self-Help Organization: Frameworks and Findings (ed TJ Powell): Chapter 13. SAGE Publications, 1994.

[16] Suresh R, Karkossa Z, Richard J, Karia M. Program evaluation of a student-led peer support service at a Canadian university. Int J Ment Health Syst 2021; 15(1): 54. 34059083 10.1186/s13033-021-00479-7 PMC8165510

[17] Equality Challenge Unit. *Equality in Higher Education: Statistical Report 2014*. Higher Education Statistics Agency (HESA), 2014 (https://www.advance-he.ac.uk/knowledge-hub/equality-higher-education-statistical-report-2014).

[18] Rickwood D, Deane FP, Wilson CJ, Ciarrochi J. Young people's help-seeking for mental health problems. Aust J Adv Ment Health 2005; 4(3): 218–51.

[19] Ebert DD, Mortier P, Kaehlke F, Bruffaerts R, Baumeister H, Auerbach RP, et al. Barriers of mental health treatment utilization among first-year college students: first cross-national results from the WHO world mental health international college student initiative. Int J Methods Psychiatr Res 2019; 28(2): e1782. 31069905 10.1002/mpr.1782 PMC6522323

[20] Bryan AE, Arkowitz H. Meta-analysis of the effects of peer-administered psychosocial interventions on symptoms of depression. Am J Community Psychol 2015; 55(3-4): 455–71. 25861883 10.1007/s10464-015-9718-y

[21] John NM, Page O, Martin SC, Whittaker P. Impact of peer support on student mental wellbeing: a systematic review. MedEdPublish 2018; 7(3): 170. 10.15694/mep.2018.0000170.1 PMC10701817 38074560

[22] Fortuna KL, Solomon P, Rivera J. An update of peer support/peer provided services underlying processes, benefits, and critical ingredients. Psychiatr Q 2022; 93(2): 571–86. 35179660 10.1007/s11126-022-09971-w PMC8855026

[23] Monk C, Purnell L. What constitutes ‘peer support’ within peer supported development? J Pedag Dev 2014; 4: 38–47.

[24] Byrom N. An evaluation of a peer support intervention for student mental health. J Ment Health 2018; 27(3): 240–6. 29451411 10.1080/09638237.2018.1437605

[25] Ferrari JR. Mentors in life and at school: impact on undergraduate prote´ge´ perceptions of university mission and values. Ment Tutor 2004; 12(3): 295–305.

[26] Jacobi M. Mentoring and undergraduate academic success: a literature review. Rev Edu Res 1991; 61(4): 505–32.

[27] Bosmans D, Young E, McLoughlin R. Does PAL work? An exploration of affect amongst first-year HE in FE students. Athens J Educ 2018; 6(1): 13–31.

[28] King AJ, Simmons MB. A systematic review of the attributes and outcomes of peer work and guidelines for reporting studies of peer interventions. Psychiatr Serv 2018; 69(9): 961–77. 29962310 10.1176/appi.ps.201700564

[29] Stoll N, Yalipende Y, Byrom NC, Hatch SL, Lempp H. Mental health and mental well-being of black students at UK universities: a review and thematic synthesis. BMJ Open 2022; 12(2): e050720. 10.1136/bmjopen-2021-050720 PMC8886426 35228276

[30] Stoll N, Yalipende Y, Haas J. *Black Students Talk x King's College London Students’ Union Report*. King's College London Students' Union (KCLSU), 2021 (https://kclpure.kcl.ac.uk/portal/en/publications/black-students-talk-x-kings-college-london-students-union-report).

[31] Brennan SE, Munn Z. PRISMA 2020: a reporting guideline for the next generation of systematic reviews. JBI Evid Synth 2021; 19(5): 906–8. 33989266 10.11124/JBIES-21-00112

[32] Page MJ, McKenzie JE, Bossuyt PM, Boutron I, Hoffmann TC, Mulrow CD, et al. The PRISMA 2020 statement: an updated guideline for reporting systematic reviews. BMJ 2021; 372: n71. 33782057 10.1136/bmj.n71 PMC8005924

[33] Campbell M, McKenzie JE, Sowden A, Katikireddi SV, Brennan SE, Ellis S, et al. Synthesis without meta-analysis (SWiM) in systematic reviews: reporting guideline. BMJ 2020; 368: l6890. 31948937 10.1136/bmj.l6890 PMC7190266

[34] OpenGrey. *OPENGREY.EU – Grey Literature Database*. The Hague: Data Archiving and Networked Services Coverage, 1980–2020 (https://opengrey.eu/).

[35] Canadian Agency for Drugs and Technologies in Health (CADTH). Grey Matters: A Tool for Searching Health-Related Grey Literature. Canadian Agency for Drugs and Technologies in Health, 2023 (https://greymatters.cadth.ca/).

[36] Upsher R, Nobili A, Hughes G, Byrom N. A systematic review of interventions embedded in curriculum to improve university student wellbeing. Educ Res R 2022; 37: 100464.

[37] Lyons N, Cooper C, Lloyd-Evans B. A systematic review and meta-analysis of group peer support interventions for people experiencing mental health conditions. BMC Psychiatry 2021; 21(1): 315. 34162340 10.1186/s12888-021-03321-z PMC8220835

[38] Hupe M. Endnote X9. J Elect Resourc Med Libraries 2019; 16(3-4): 117–9.

[39] Ouzzani M, Hammady H, Fedorowicz Z, Elmagarmid A. Rayyan—a web and mobile app for systematic reviews. Syst Rev 2016; 5(1): 210. 27919275 10.1186/s13643-016-0384-4 PMC5139140

[40] National Heart, Lung, and Blood Institute (NHLBI). *Study Quality Assessment Tools 2021*. NHLBI, 2021 (https://www.nhlbi.nih.gov/health-topics/study-quality-assessment-tools).

[41] StataCorp. Stata Statistical Software: Release 17. StataCorp LLC, 2021 (https://www.stata.com/).

[42] McKenzie JE, Brennan SE. Synthesizing and presenting findings using other methods. In Cochrane Handbook for Systematic Reviews of Interventions: 321–47. Wiley, 2019.

[43] Cohen S, Kessler RC, Gordon LU. Measuring Stress: A Guide for Health and Social Scientists. Oxford University Press, 1995.

[44] Chipas A, McKenna D. Stress and burnout in nurse anesthesia. AANA J 2011; 79(2): 122–8. 21560975

[45] Lovibond SH. P.F. Manual for the Depresession Anxiety Stress Scales. Psychology Foundation, 1995.

[46] House RJ, Rizzo JR. Role conflict and ambiguity as critical variables in a model of organizational behavior. Organ Behav Hum Perform 1972; 7(3): 467–505.

[47] Allen TD, McManus SE, Russell JEA. Newcomer socialization and stress: formal peer relationships as a source of support. J Vocat Behav 1999; 54(3): 453–70.

[48] Spielberger CD. State-Trait Anxiety Inventory. The Corsini Encyclopedia of Psychology. John Wiley & Sons, 2010.

[49] Spitzer RL, Kroenke K, Williams JBW, Löwe B. A brief measure for assessing generalized anxiety disorder: the GAD-7. Arch Intern Med 2006; 166(10): 1092–7. 16717171 10.1001/archinte.166.10.1092

[50] Caballo V, Salazar I, Arias B, Irurtia M, Calderero M, Graña J, et al. Validation of the social anxiety questionnaire for adults (SAQ-A30) with Spanish university students: similarities and differences among degree subjects and regions. Behav Psychol Psicol Conduc 2010; 18: 5–34.

[51] Liebowitz MR. Psychopharmacological management of social and simple phobias. In The Clinical Management of Anxiety Disorders (eds W Coryell, G Winokur): 63–78. Oxford University Press, 1991.

[52] Reynolds CR, Richmond BO, Lowe PA. The Adult Manifest Anxiety Scale. Western Psychological Services, 2003.

[53] Beck AT, Steer RA, Brown GK. Beck Depression Inventory-II. Psychological Corporation, 1996.

[54] Kohout FJ, Berkman LF, Evans DA, Cornoni-Huntley J. Two shorter forms of the CES-D (Center For Epidemiological Studies Depression) depression symptoms index. J Aging Health 1993; 5(2): 179–93. 10125443 10.1177/089826439300500202

[55] Martin CR, Redshaw M. Establishing a coherent and replicable measurement model of the Edinburgh Postnatal Depression Scale. Psychiatry Res 2018; 264: 182–91. 29649675 10.1016/j.psychres.2018.03.062 PMC6008486

[56] Adewuya AO, Ola BA, Afolabi OO. Validity of the Patient Health Questionnaire (PHQ-9) as a screening tool for depression amongst Nigerian university students. J Affect Disord 2006; 96(1–2): 89–93. 16857265 10.1016/j.jad.2006.05.021

[57] Stewart-Brown S, Tennant A, Tennant R, Platt S, Parkinson J, Weich S. Internal construct validity of the Warwick–Edinburgh Mental Well-Being Scale (WEMWBS): a Rasch analysis using data from the Scottish health education population survey. Health Qual Life Outcomes 2009; 7(1): 15. 19228398 10.1186/1477-7525-7-15 PMC2669062

[58] Tennant R, Hiller L, Fishwick R, Platt S, Joseph S, Weich S, et al. The Warwick–Edinburgh Mental Well-Being Scale (WEMWBS): development and UK validation. Health Qual Life Outcomes 2007; 5(1): 63. 18042300 10.1186/1477-7525-5-63 PMC2222612

[59] Gençöz T. Positive and Negative Affect Schedule: a study of validity and reliability. Turk J Psychol 2000; 46: 19–26.

[60] Watson D, Clark LA, Tellegen A. Development and validation of brief measures of positive and negative affect: the PANAS scales. J Pers Soc Psychol 1988; 54(6): 1063–70. 3397865 10.1037//0022-3514.54.6.1063

[61] Diener E. Assessing subjective well-being: progress and opportunities. Soc Indic Res 1994; 31(2): 103–57.

[62] Koker S. Yayinlanmamis yuksek lisans tezi. [Comparison of life satisfaction among normal and problematic adolescents]. *Unpublished master's thesis*. Institute of Social Sciences, Ankara University, 1991.

[63] Russell D, Peplau LA, Cutrona CE. The revised UCLA loneliness scale: concurrent and discriminant validity evidence. J Pers Soc Psychol 1980; 39(3): 472–80. 7431205 10.1037//0022-3514.39.3.472

[64] Evans C, Connell J, Barkham M, Margison F, McGrath G, Mellor-Clark J, et al. Towards a standardised brief outcome measure: psychometric properties and utility of the CORE-OM. Br J Psychiatry 2002; 180: 51–60. 11772852 10.1192/bjp.180.1.51

[65] Derogatis LR, Melisaratos N. The brief symptom inventory: an introductory report. Psychol Med 1983; 13(3): 595–605. 6622612

[66] Goldberg D, Williams PA. A User's Guide to the General Health Questionnaire. NFER-Nelson Publishing Company, 1988.

[67] Campbell A, Converse PE, Rodgers WL. The Quality of American Life: Perceptions, Evaluations and Satisfaction. Russell Sage Foundation, 1976.

[68] Crawford JR, Henry JD. The Positive and Negative Affect Schedule (PANAS): construct validity, measurement properties and normative data in a large non-clinical sample. Br J Clin Psychol 2004; 43(Pt 3): 245–65. 15333231 10.1348/0144665031752934

[69] Fairburn CG, Beglin SJ. Assessment of eating disorders: interview or self-report questionnaire? Int J Eat Disord 1994; 16(4): 363–70. 7866415

[70] Wagnild GM, Young HM. Development and psychometric evaluation of the resilience scale. J Nurs Meas 1993; 1(2): 165–78. 7850498

[71] Flugel Colle KF, Vincent A, Cha SS, Loehrer LL, Bauer BA, Wahner-Roedler DL. Measurement of quality of life and participant experience with the mindfulness-based stress reduction program. Complement Ther Clin Pract 2010; 16(1): 36–40. 20129408 10.1016/j.ctcp.2009.06.008

[72] Pavot W, Diener E. Review of the satisfaction with life scale. Psychol Assess 1993; 5: 164–72.

[73] Cutrona C, Russell D. The provisions of social relationships and adaptation to stress. In Advances in Personal Relationships, Vol. 1 (eds W Jones, D Perlman): 37–67. JAI Press, 1987.

[74] Lambert MJ, Hansen N, Umphress V, Lunnen K, Okiishi J, Burlingame G, et al. Administration and Scoring Manual for the Outcome Questionnaire (OQ-45.2). Wilmington, DE: American Professional Credentialing Services, 1996.

[75] French BF, Oakes WC. Reliability and validity evidence for the Institutional Integration Scale. Educ Psychol Meas 2004; 64(1): 88–98.

[76] Sherer M, Maddux JE, Mercandante B, Prentice-Dunn S, Jacobs B, Rogers RW. The Self-Efficacy Scale: construction and validation. Psychol Rep 1982; 51: 663–71.

[77] Tipton RM, Worthington JR. The measurement of generalized self-efficacy: a study of construct validity. J Pers Assess 1984; 48(5): 545–8. 16367514 10.1207/s15327752jpa4805_14

[78] Rosenberg M. Society and the Adolescent Self-Image. Princeton University Press, 1965.

[79] Koenig-Lewis N, Palmer A, Dermody J, Urbye A. Consumer's evaluations of ecological packaging – rational and emotional approaches. J Environ Psychol 2014; 37: 94–105.

[80] Esses VM, Burstein M, Ravanera Z, Hallman S, Medianu S. Alberta Settlement Outcomes Survey. Pathways to Prosperity Partnership, 2013 (https://open.alberta.ca/publications/alberta-settlement-outcomes-survey).

[81] Sandhu DS, Asrabadi BR. Development of an acculturative stress scale for international students: preliminary findings. Psychol Rep 1994; 75(1 Pt 2): 435–48. 7809315 10.2466/pr0.1994.75.1.435

[82] Yang B, Clum GA. Measures of life stress and social support specific to an Asian student population. J Psychopathol Behav Assess 1995; 17(1): 51–67.

[83] Wei M, Wang KT, Ku TY. A development and validation of the perceived language discrimination scale. Cultur Divers Ethnic Minor Psychol 2012; 18(4): 340–51. 22866690 10.1037/a0029453

[84] Burmeister G. Changes in anxiety levels in mature nursing students with peer dyad use during the clinical experience. Doctor of Nursing Practice doctoral thesis Marybelle and S. Paul Musco School of Nursing and Health Professions, Brandman University, 2016.

[85] Collings R, Swanson V, Watkins R. The impact of peer mentoring on levels of student wellbeing, integration and retention: a controlled comparative evaluation of residential students in UK higher education. Higher Educ 2014; 68(6): 927–42.

[86] Conley CS, Hundert CG, Charles JLK, Huguenel BM, Al-khouja M, Qin S, et al. Honest, open, proud-college: effectiveness of a peer-led small-group intervention for reducing the stigma of mental illness. Stigma Health 2020; 5(2): 168–78.

[87] Eren-Sisman EN, Cigdemoglu C, Geban O. The effect of peer-led team learning on undergraduate engineering students’ conceptual understanding, state anxiety, and social anxiety. Chem Educ Res Pract 2018; 19(3): 694–710.

[88] Eryilmaz A. The effectiveness of a peer-helping programme that increases subjective well-being. Br J Guid Couns 2017; 45(3): 225–37.

[89] Fontana AM, Hyra D, Godfrey L, Cermak L. Impact of a peer-led stress inoculation training intervention on state anxiety and heart rate in college students. J Appl Biobehav Res 1999; 4(1): 45–63.

[90] Freeman E, Barker C, Pistrang N. Outcome of an online mutual support group for college students with psychological problems. Cyberpsychol Behav 2008; 11(5): 591–3. 18817485 10.1089/cpb.2007.0133

[91] Frohn AF, Turecka S, Katz J. Can informal peer education help college students manage stress? effects of two brief skills-based programs on student stress, rumination, and self-criticism. In Psychology of Well-Being: Theory, Perspectives and Practice (ed E Noehammer): 19–31. NovaScience Publishers, 2013.

[92] Fullick JM, Smith-Jentsch KA, Yarbrough CS, Scielzo SA. Mentor and protege goal orientations as predictors of newcomer stress. J Sch Teach Learn 2012; 12(1): 59–73.

[93] Geng G, Midford R, Buckworth J, Kersten T. Tapping into the teaching experiences of final year education students to increase support for students in their first year. Stud Success 2017; 8(1): 13–23.

[94] Head EG. The use of peer mentoring to decrease stress in student registered nurse anesthetists. Doctor of Nursing Practice doctoral thesis Department of Advanced Practice, University of Southern Mississippi, 2015.

[95] Humphrey KR. Using a student-led support group to reduce stress and burnout among BSW students. Soc Work With Groups 2013; 36(1): 73–84.

[96] Hwang WC, Chan CP. Compassionate meditation to heal from race-related stress: a pilot study with Asian Americans. Am J Orthopsychiatry 2019; 89(4): 482–92. 31305116 10.1037/ort0000372

[97] Kilpela LS, Blomquist K, Verzijl C, Wilfred S, Beyl R, Becker CB. The body project 4 all: a pilot randomized controlled trial of a mixed-gender dissonance-based body image program. Int J Eat Disord 2016; 49(6): 591–602. 27188688 10.1002/eat.22562 PMC5365075

[98] Kocak R. The effects of cooperative learning on psychological and social traits among undergraduate students. Soc Behav Pers 2008; 36(6): 771–82.

[99] Mattanah JF, Ayers JF, Brand BL, Brooks LJ, Quimby JL, McNary SW. A social support intervention to ease the college transition: exploring main effects and moderators. J Coll Stud Dev 2010; 51(1): 93–108.

[100] Mattanah JF, Brooks LJ, Brand BL, Quimby JL, Ayers JF. A social support intervention and academic achievement in college: does perceived loneliness mediate the relationship? J Coll Couns 2012; 15(1): 22–36.

[101] McNulty K. The effects of peer mentoring on the stress levels of nursing students. Doctor of Education doctoral thesis College of Saint Mary, 2018.

[102] Moir F, Henning M, Hassed C, Moyes SA, Elley CR. A peer-support and mindfulness program to improve the mental health of medical students. Teach Learn Med 2016; 28(3): 293–302. 27092397 10.1080/10401334.2016.1153475

[103] Petersen TJ. Evaluation of a stress management program for newly matriculated first-generation college students: a randomized controlled trial. Doctor of Philosophy doctoral thesis Department of Clinical Psychology, Ohio University, 2013.

[104] Pfister VR. Effects of faculty and peer mentoring on perceived stress and social support of college student athletes. Doctor of Philosophy doctoral thesis Department of Adult, Career and Higher Education, University of South Florida, 2004.

[105] Phinney JS, Torres Campos CM, Padilla Kallemeyn DM, Kim C. Processes and outcomes of a mentoring program for Latino college freshmen. J Soc Issues 2011; 67(3): 599–621.

[106] Pinks D, Warren-James M, Katsikitis M. Does a peer social support group intervention using the cares skills framework improve emotional expression and emotion-focused coping in paramedic students? Australas Emerg Care 2021; 24: 308–13. 10.1016/j.auec.2021.03.005 33836985

[107] Short E, Kinman G, Baker S. Evaluating the impact of a peer coaching intervention on well-being amongst psychology undergraduate students. Int Coach Psychol Rev 2010; 5: 27–35.

[108] Siew CT, Mazzucchelli TG, Rooney R, Girdler S. A specialist peer mentoring program for university students on the autism spectrum: a pilot study. PLoS One 2017; 12(7): e0180854. 10.1371/journal.pone.0180854 PMC5509180 28704446

[109] Thomson C, Esses VM. Helping the transition: mentorship to support international students in Canada. J Int Stud 2016; 6(4): 873–86.

[110] Noe RA. An investigation of the determinants of successful assigned mentoring relationships. Pers Psychol 1988; 41(3): 457–79.

[111] Gall TL, Evans DR, Bellerose S. Transition to first-year university: patterns of change in adjustment across life domains and time. J Soc Clin Psychol 2000; 19(4): 544.

[112] Conley CS, Shapiro JB, Huguenel BM, Kirsch AC. Navigating the college years: developmental trajectories and gender differences in psychological functioning, cognitive-affective strategies, and social well-being. Emerg Adulthood 2020; 8(2): 103–17.

[113] Bewick B, Koutsopoulou G, Miles J, Slaa E, Barkham M. Changes in undergraduate students’ psychological well-being as they progress through university. Stud Higher Educ 2010; 35(6): 633–45.

[114] MacLean L, Booza J, Balon R. The impact of medical school on student mental health. Acad Psychiatry 2016; 40(1): 89–91. 25749920 10.1007/s40596-015-0301-5

[115] Jacob R, Li T-y, Martin Z, Burren A, Watson P, Kant R, et al. Taking care of our future doctors: a service evaluation of a medical student mental health service. BMC Med Educ 2020; 20(1): 172. 32471406 10.1186/s12909-020-02075-8 PMC7257172

[116] Ruiz-Hernández JA, Guillén Á, Pina D, Puente-López E. Mental health and healthy habits in university students: a comparative associative study. Eur J Investig Health Psychol Educ 2022; 12(2): 114–26. 10.3390/ejihpe12020010 PMC8871110 35200233

[117] Reverté-Villarroya S, Ortega L, Raigal-Aran L, Sauras-Colón E, Ricomà-Muntané R, Ballester-Ferrando D, et al. Psychological well-being in nursing students: a multicentric, cross-sectional study. Int J Environ Res Public Health 2021; 18(6): 3020. 10.3390/ijerph18063020 PMC7999566 33804156

[118] Davidson L, Chinman M, Kloos B, Weingarten R, Stayner D, Kraemer Tebes J. Peer support among individuals with severe mental illness: a review of the evidence. Clin Psychol 1999; 6(2): 165–87.

[119] Parsloe E, Wray M. Coaching and Mentoring: Practical Methods to Improve Learning. Kogan Page Publishers, 2000.

[120] Budge S. Peer mentoring in postsecondary education: implications for research and practice. J Coll Read Learn 2006; 37(1): 71–85.

[121] Johnson DW, Johonson RT. Cooperation and Competition: Theory and Research. Interacting Book Company, 1989.

[122] Lee C, Ng M, Jacobs G. Cooperative learning in the thinking classroom: research and theoretical perspectives. (1–6 Jun 1997) *7th International Conference on Thinking, Singapore*. Thinking School Learning Nation, 1997.

[123] Tien LT, Roth V, Kampmeier JA. Implementation of a peer-led team learning instructional approach in an undergraduate organic chemistry course. J Res Sci Teach 2002; 39(7): 606–32.

[124] Varma-Nelson P. Peer-led team learning. Metropolitan Universities J 2006; 7(4): 19–29.

[125] Varma-Nelson P, Coppola B, Greenbowe T, Pienta N. Team learning. In The Chemists’ Guide to Effective Teaching. 1 (ed Cooper MM): 155–69. Prentice Hall Publishing, 2005.

[126] University of Bournemouth. *PAL Academic Course Contact Guide: What Is PAL?*. University of Bournemouth, 2022 (https://libguides.bournemouth.ac.uk/c.php?g=661264&p=4671223).

[127] Hilsdon J. Peer learning for change in higher education. Innov Educ Teach Int 2014; 51(3): 244–54.

[128] Ody M, Carey W. Peer education. In The Student Engagement Handbook: Practice in Higher Education (eds E Dunne, D Owen): 291. Emerald Group Publishing, 2013.

[129] Pfeiffer PN, Heisler M, Piette JD, Rogers MA, Valenstein M. Efficacy of peer support interventions for depression: a meta-analysis. Gen Hosp Psychiatry 2011; 33(1): 29–36. 21353125 10.1016/j.genhosppsych.2010.10.002 PMC3052992

[130] Andrews J, Clark R, Davies K. Peer Mentoring Works! How Peer Mentoring Enhances Student Success in Higher Education: Evaluation Toolkit. Higher Education Academy, 2011 (https://research.aston.ac.uk/en/publications/peer-mentoring-works-how-peer-mentoring-enhances-student-success-).

[131] Araujo N. Reviewing the evidence of effective peer education among young people. Perspect Public Health 2018; 138(6): 299–300. 30412024 10.1177/1757913918801472

[132] Biggers M, Yilmaz T, Sweat M. Using collaborative, modified peer-led team learning to improve student success and retention in intro cs. *40th ACM Technical Symposium on Computer Science Education (Tennessee, USA, 4–7 Mar 2009).* Association for Computing Machinery, 2009.

[133] Paez A. Grey literature: an important resource in systematic reviews. J Evid Based Med 2017; 10(3): 233–40. 28857505 10.1111/jebm.12266

[134] Groves T. Enhancing the quality and transparency of health research. BMJ 2008; 337(7661): a718. 10.1136/bmj.a718 PMC2453250 18614488

[135] Schulz KF, Altman DG, Moher D. CONSORT 2010 statement: updated guidelines for reporting parallel group randomised trials. J Pharmacol Pharmacother 2010; 1(2): 100–7. 21350618 10.4103/0976-500X.72352 PMC3043330

[136] Dodd AL, Priestley M, Tyrrell K, Cygan S, Newell C, Byrom NC. University student well-being in the United Kingdom: a scoping review of its conceptualisation and measurement. J Ment Health 2021; 30(3): 375–87. 33567937 10.1080/09638237.2021.1875419

[137] Broglia E, Nisbet K, Chow H, Bone C, Simmonds-Buckley M, Knowles L, et al. Student Services Partnerships Evaluation And Quality Standards (SPEQS) Toolkit. UWE Bristol, 2022 (https://www.uwe.ac.uk/-/media/uwe/documents/about/speqs-toolkit.pdf).

[138] Borenstein M, Hedges LV, Higgins JPT, Rothstein HR. Meta-analysis methods based on direction and *P*-values. In Introduction to Meta-Analysis (eds M Borenstein, LV Hedges, JPT Higgins, HR Rothstein): 325–30. John Wiley & Sons, 2009.

[139] Higgins JP, Thomas J, Chandler J, Cumpston M, Li T, Page MJ, et al. Cochrane Handbook for Systematic Reviews of Interventions. John Wiley & Sons, 2019. 10.1002/14651858.ED000142 PMC10284251 31643080

