# A systematic review of peer support interventions for student mental health and well-being in higher education – CORRIGENDUM

**DOI:** 10.1192/bjo.2024.800

**Published:** 2024-09-18

**Authors:** Julia Pointon-Haas, Luqmaan Waqar, Rebecca Upsher, Juliet Foster, Nicola Byrom, Jennifer Oates

This article was originally published with an error stating ‘Correspondingly, British HEIs reported a 94% increase in demand for counselling services from 2012 to 2017’. This should have read ‘Correspondingly, 94% of British HEIs reported an increase in demand for counselling services from 2012–2017.’

This has now been updated and this corrigendum published.
