# Supplementary material for: A systematic review of peer support interventions for student mental health and well-being in higher education
Source: BJPsych Open. 2023 Dec 15;10(1):e12. doi: 10.1192/bjo.2023.603 (PMC10755562; doi:10.1192/bjo.2023.603)
Supplement: Pointon-Haas et al. supplementary material [file S2056472423006038sup001.docx]

**Supplementary tables**

**Table S1**

*Database: APA PsycInfo (OVID) <1806 to May Week 2 2021>*

*Database: Embase (OVID) <1974 to 2021 Week 19>*

*Database: Ovid MEDLINE(R) and Epub Ahead of Print, In-Process, In-Data-Review & Other Non-Indexed Citations and Daily <1946 to May 18, 2021>*

**Search Strategy:**

--------------------------------------------------------------------------------

1     exp MENTAL HEALTH/

2     "psychological outcome$".ti,ab.

3     exp mood disorders/

4     exp DEPRESSION/

5     Wellbeing.ti,ab.

6     "psychological well being".ti,ab.

7     "mental well being".ti,ab.

8     exp social anxiety/ or exp ANXIETY/ or anxiety disorders/ or stress.mp.

9     exp Psychological Stress/ or exp "Resilience (Psychological)"/

10     exp Well Being/ or Well?being.mp. or exp wellness/ or wellness.mp.

11     exp Belonging/ or Belong*.mp.

12     exp loneliness/ or loneliness.mp.

13     or/1-12

14     (peersupport or peer support or peer to peer or p2p or selfhelp or self-help or social network or social support or support network or support group or support system).ab,ti.

15     (student* adj3 (led* or run*)).mp. [mp=title, abstract, heading word, drug trade name, original title, device manufacturer, drug manufacturer, device trade name, keyword, floating subheading word, candidate term word]

16     "peer-assisted learning".mp.

17     exp Cooperative Learning/ or exp Peer Tutoring/

18     exp Mentor/ or exp peer mentor/

19     exp Peers/ or exp Peer Relations/ or exp Social Support/

20     "peer health education".mp.

21     (Peer* adj3 (support* or led or lead* or deliver* or run* or held or direct* or online or online or forum*)).mp.

22     ((lay or layperson* or laypeople or lay people) adj3 (support* or led or lead* or deliver* or run* or held or direct* or online or on line or forum*)).mp.

23     (Paraprofessional* adj3 (support* or led or lead* or deliver* or run* or held or direct* or online or on line or forum*)).mp.

24     (mutual* adj2 (aid* or support* or help*)).ti,ab.

25     or/14-24

26     college.mp. or university.ti,ab.

27     exp undergraduates/

28     exp Postgraduate Students/

29     students.mp. and ((college* or universit*).tw. or (college* or universit*).ti,ab.)

30     young adult*.ab. and ((college* or universit*).tw. or (college* or universit*).ti,ab.)

31     older adolescent*.mp. and ((college* or universit*).tw. or (college* or universit*).ti,ab.)

32     university-based.af.

33     ((student* or faculty or academic or postgraduate or graduate or professional* or school*) adj5 (universit* or college or tertiary or "higher education" or campus)).tw.

34     exp universities/ or exp college/

35     or/26-34

36     13 and 25 and 35

37     limit 36 to yr="1991 -Current

| **Table S2** | | | | | | | | | | | | | |
| --- | --- | --- | --- | --- | --- | --- | --- | --- | --- | --- | --- | --- | --- |
| ***Risk of Bias Scoring*** | | | | | | | | | | | | | |
|  | **1** | **2** | **3** | **4** | **5** | **6** | **7** | **8** | **9** | **10** | **11** | **12** | **Rating** |
| **Paper** | Clarity of Objectives | Clarity of Eligibility Criteria | Representativeness | Entry Criteria | Sample Size | Intervention Description | Outcome Measures | Blinded | Attrition Rate | Statistical Values | Multiple Time Points | Individual-level data controlled | Quality Rating |
| (Bosmans et al., 2018) [1] | Yes | Yes | Yes | Yes | NR | Yes | Yes | NA | CD | Yes | No | NA | Fair |
| (Burmeister, 2017) [2] | Yes | Yes | Yes | Yes | NR | Yes | Yes | NA | Yes | Yes | No | NA | Good/Fair |
| (Byrom, 2018) [3] | Yes | Yes | Yes | Yes | Yes | Yes | Yes | NA | No | Yes | Yes | NA | Good |
| (Collings et al., 2014) [4] | Yes | Yes | Yes | Yes | NR | Yes | Yes | NA | No | Yes | No | NA | Fair |
| (Conley et al., 2020) [5] | Yes | Yes | Yes | Yes | Yes | Yes | Yes | NA | Yes | Yes | Yes | NA | Good |
| (Eren-Sisman et al., 2018) [6] | Yes | Yes | Yes | Yes | NR | Yes | Yes | NA | Yes | Yes | No | NA | Fair |
| (Eryilmaz, 2017) [7] | Yes | Yes | Yes | Yes | NR | Yes | Yes | NA | Yes | Yes | Yes | NA | Good/Fair |
| (Fontana et al., 1999) [8] | Yes | Yes | Yes | Yes | NR | Yes | Yes | NA | Yes | Yes | Yes | NA | Good/Fair |
| (Freeman et al., 2008) [9] | Yes | Yes | Yes | Yes | NR | Yes | Yes | NA | No | Yes | No | NA | Fair |
| (Frohn et al., 2013) [10] | Yes | Yes | Yes | Yes | NR | Yes | Yes | NA | Yes | Yes | No | NA | Fair |
| (Fullick et al., 2012) [11] | Yes | Yes | Yes | Yes | NR | Yes | Yes | NA | NR | Yes | No | NA | Poor |
| (Geng et al., 2017) [12] | Yes | Yes | Yes | Yes | No | Yes | Yes | NA | Yes | No | Yes | NA | Poor |
| (Head, 2016) [13] | Yes | Yes | Yes | Yes | No | Yes | Yes | NA | No | Yes | No | NA | Fair |
| (Humphrey, 2013) [14] | Yes | Yes | Yes | Yes | NR | Yes | Yes | NA | CD | CD | No | NA | Poor |
| (Hwang & Chan, 2019) [15] | Yes | Yes | Yes | Yes | NR | Yes | Yes | NA | Yes | Yes | No | NA | Fair |
| (Kilpela et al., 2016),[16] | Yes | Yes | Yes | Yes | Yes | Yes | Yes | NA | Yes | No | Yes | NA | Fair |
| (Kocak, 2008) [17] | Yes | Yes | Yes | Yes | NR | Yes | Yes | NA | Yes | Yes | No | NA | Fair |
| (Mattanah et al., 2010) [18] | Yes | Yes | Yes | Yes | Yes | Yes | Yes | NA | No | Yes | Yes | NA | Good |
| (Mattanah et al., 2012) [19] | Yes | Yes | Yes | Yes | Yes | Yes | Yes | NA | No | No | No | NA | Fair/poor |
| (McNulty, 2018), [20] | Yes | Yes | Yes | Yes | NR | Yes | Yes | NA | Yes | No | No | NA | Fair |
| (Moir et al., 2016)[21] | Yes | Yes | Yes | Yes | Yes | Yes | Yes | NA | Yes | Yes | No | NA | Good |
| (Petersen, 2015) [22] | Yes | Yes | Yes | Yes | CD | Yes | Yes | NA | Yes | Yes | No | NA | Good |
| (Pfister, 2006) [23] | Yes | Yes | Yes | Yes | NR | Yes | Yes | NA | Yes | Yes | Yes | NA | Good/Fair |
| (Phinney et al., 2011) [24] | Yes | Yes | Yes | Yes | NR | Yes | Yes | NA | NR | CD | No | NA | Poor |
| (Pinks et al., 2021) [25] | Yes | Yes | Yes | Yes | NR | Yes | Yes | NA | No | Yes | No | NA | Poor |
| (Short et al., 2010) [26] | Yes | Yes | Yes | Yes | NR | Yes | Yes | NA | NR | Yes | No | NA | Poor |
| (Siew et al., 2017) [27] | Yes | Yes | Yes | Yes | NR | Yes | Yes | NA | NR | Yes | No | NA | Fair |
| (Thomson & Esses, 2016) [28] | Yes | Yes | Yes | Yes | NR | Yes | Yes | NA | Yes | Yes | No | NA | Fair |

| **Table S3** | |
| --- | --- |
| *Acronyms & Definitions of Mental Health & Wellbeing Measures* | |
| AMAS-C | Adult Manifest Anxiety Scale-College Version |
| ASSIS | Acculturative Stress Scale for International Students |
| BDI-II | Beck Depression Inventory |
| BSI | Brief Symptom Inventory |
| CES-D | Center for Epidemiologic Studies Depression Scale |
| CORE-OM | CORE Outcome Measure |
| DASS-21 | Depression, Anxiety and Stress Scale |
| EDE-Q | Eating Disorder Examination Questionnaire |
| EPDS | Edinburgh Postnatal Depression Scale |
| GAD-7 | Generalised Anxiety Disorder |
| GHQ-12 | General Health Questionnaire |
| LASA | Linear Analogue Self-Assessment |
| LSAS | Liebowitz Social Anxiety Scale |
| OQ-45.2 | Outcomes Questionnaire |
| PANAS | Positive and Negative Affect Schedule |
| PHQ-9 | Primary Health Questionnaire |
| PSS | Perceived Stress Scale |
| RES | Rosenberg’s (1965) Self Esteem Scale |
| RS15 | Resilience |
| R-UCLA Loneliness | Revised University of California-Los Angeles Loneliness Scale |
| SAQ | Social Anxiety Questionnaire for Adult |
| SPS | Social Provisions Scale |
| STAI | State-Trait Anxiety Inventory |
| SWEMWBS | Shortened Warwick-Edinburgh Scale of Wellbeing |
| SWLS | Satisfaction with Life Scale |
| UCLA Loneliness | University of California-Los Angeles Loneliness Scale |
